# Effects of spatiotemporal HSV-2 lesion dynamics and antiviral treatment on the risk of HIV-1 acquisition

**DOI:** 10.1371/journal.pcbi.1006129

**Published:** 2018-04-26

**Authors:** Catherine M. Byrne, Soren Gantt, Daniel Coombs

**Affiliations:** 1 Department of Microbiology and Immunology, University of British Columbia, Vancouver, British Columbia, Canada; 2 Institute of Applied Mathematics, University of British Columbia, Vancouver, British Columbia, Canada; 3 British Columbia Children’s Hospital, Vancouver, British Columbia, Canada; 4 Department of Mathematics, University of British Columbia, Vancouver, British Columbia, Canada; ETH Zurich, SWITZERLAND

## Abstract

Patients with Herpes Simplex Virus-2 (HSV-2) infection face a significantly higher risk of contracting HIV-1. This is thought to be due to herpetic lesions serving as entry points for HIV-1 and tissue-resident CD4+ T cell counts increasing during HSV-2 lesional events. We have created a stochastic and spatial mathematical model describing the dynamics of HSV-2 infection and immune response in the genital mucosa. Using our model, we first study the dynamics of a developing HSV-2 lesion. We then use our model to quantify the risk of infection with HIV-1 following sexual exposure in HSV-2 positive women. Untreated, we find that HSV-2 infected women are up to 8.6 times more likely to acquire HIV-1 than healthy patients. However, when including the effects of the HSV-2 antiviral drug, pritelivir, the risk of HIV-1 infection is predicted to decrease by up to 35%, depending on drug dosage. We estimate the relative importance of decreased tissue damage versus decreased CD4+ cell presence in determining the effectiveness of pritelivir in reducing HIV-1 infection. Our results suggest that clinical trials should be performed to evaluate the effectiveness of pritelivir or similar agents in preventing HIV-1 infection in HSV-2 positive women.

## Author summary

The risk of contracting HIV-1 is significantly higher in people who have genital HSV-2 infections. Here, we put forward a new mathematical model to describe HSV-2 infection and the process of HIV-1 infection in the genital mucosa surrounding HSV-2 lesions. We determine how the characteristics of HSV-2 infection affect the risk of HIV-1 infection, and determine whether reducing the severity of HSV-2 symptoms with antiviral drugs can be expected to decrease the risk of HIV-1 infection. We find that the risk of HIV-1 infection is dependent on three factors: the amount of HIV-1 the patient is exposed to, the severity of HSV-2 lesions, and the number of CD4+ T immune cells in the genital mucosa. Our model predicts that antiviral drugs targeting HSV-2 can cause a therapeutic decrease in lesion severity and CD4+ T cell count in the genital mucosa. This furthermore causes a significant decrease in the risk of HIV-1 infection but the dose of HSV-2 antiviral drug must be sufficiently high. Our results support further development and testing of new HSV-2 antiviral drugs to help decrease the world-wide burden of HIV-1.

## Introduction

Herpes simplex virus-2 (HSV-2) is one of the most common sexually transmitted infections (STIs). Estimates from 2012 indicate that around 20 million people are newly infected by HSV-2 every year, with 11.3% of the human population infected [1]. While STIs often coincide, the establishment of human immunodeficiency virus 1 (HIV-1) in HSV-2 infected individuals is shockingly common. An estimated 38-60% of new HIV-1 infections in women and 8-49% of new HIV-1 infections in men may be attributable to HSV-2 infection due to the enhanced conditions a herpetic genital lesion presents for the entry and establishment of HIV-1 [2–4].

Genital HSV-2 lesions compromise the natural barrier of the skin and facilitate entry of HIV-1. In addition, the tissue surrounding a herpetic lesion is often rich in CD4+ T cells, the main target cell for HIV-1. These conditions may cause a 2 to 3-fold increase in the probability of HIV-1 infection establishment and drastically increase the spread of the HIV-1 epidemic [2, 5]. For this reason, it is important to understand the relationship between HSV-2 and HIV-1 infections and to find ways to decrease the risk of HSV-2 positive patients acquiring an HIV-1 infection.

The spread of HSV-2 usually occurs through skin-to-skin genital contact where the virus infects epithelial cells and replicates within them. Following this initial infection, the virus spreads to nearby neurons where it establishes latency in the dorsal roots of the neural ganglions [6]. This reservoir of HSV-2 in nerve tissue is protected from the immune system, leading to life-long infection. Viruses are slowly shed from the neurons and released back into the genital tract where they may spark a new productive epithelial infection, viral shedding, and ultimately transmission during sexual contact [7].

The development of a herpetic lesion in the epithelial tissue is largely dependent on the immune presence at that site [7]. Despite the high number of shedding episodes that occur in HSV-2 positive patients, the immune system rapidly responds, clearing small plaques of infection in two to twelve hours [8]. The two types of immune cells thought to be most important in HSV-2 infection control are the CD4+ and CD8+ T cells. Once the lesion is resolved, these immune cells also prevent re-infection, remaining at previous sites of infection for up to twenty weeks [9].

Cytotoxic CD8+ T cells are often thought of as the main effector cell population responsible for the control of HSV-2 in the infected epithelium [10]. As such, previous mathematical models of HSV-2 have included CD8+ T cells as the only immune cell present during HSV-2 infections [7, 9, 11–13]. However, CD4+ T cells have more recently been shown to be important in the control of HSV. In experiments where CD8+ T cell deficient mice were infected with HSV-1, the virus and lesions could still be cleared at genital and neural sites. However, in the alternative situation where CD4+ T cell deficient mice were infected with HSV-1, the infection could not be cleared [14]. CD4+ T cells are among the first immune cells to arrive at the site of infection, appearing within the first 48 hours of HSV-2 infection in the epithelium [15]. Once CD4+ T cells arrive, they release interferon gamma (IFN-*γ*) and other cytokines required for CD8+ T cell recruitment to the infection site [16]. Activated CD8+ T cells then kill infected cells by delivering perforin and activating apoptotic pathways [17]. Thus, CD4+ T cell dynamics should be included in mathematical models of HSV-2 infection to fully represent the system.

The influx of immune cells to the lesion site creates a favourable environment for HIV-1. Not only does HIV-1 have a greater probability of successfully traversing the damaged epithelial layer at the lesion site, but the immune response creates an environment dense in CD4+ T cells, the primary target cell of HIV-1 [2, 18]. Additionally, CD4+ T cells at lesions express high levels of chemokine receptor type 5 (CCR5), the co-receptor which HIV-1 most commonly uses to establish initial infection [4]. *Ex-vivo* studies have also shown a strong effect of HSV-2 infection on HIV-1 dynamics at herpetic lesions. In cervical tissue cultures infected with HSV-2, HIV-1 virions attached more frequently to sites containing HSV-2 infected cells than to sites containing uninfected epithelial cells, indicating that HIV-1 preferentially establishes infection at areas of HSV-2 infection [3].

HSV-2 and HIV-1 coinfection can also lead to higher transmission of HIV-1, with genital ulcers or microlesions shedding both HIV-1 and HSV-2 [19]. With 80% of HSV-2 viral shedding events occurring without visible lesions, this creates a potentially significant transmission scenario for both viruses [8]. Without proper knowledge about the state of their infection, infected individuals may remain asymptomatic and unknowingly infect sexual partners with either virus. Reciprocally, an asymptomatic HSV-2 positive individual may unknowingly be at an increased risk of HIV-1.

While no drug or vaccine has been developed that is capable of completely clearing or preventing HSV-2 infection, antivirals designed to decrease infection severity and outbreak frequency have long been available. Acyclovir and other related compounds work by inhibiting HSV-specific DNA polymerases and helicases, and result in less viral replication [20]. Acyclovir and its ester prodrug, valacyclovir, have been shown to reduce the occurrence of genital lesions by 47-75% and the rate of viral shedding by 80% [21, 22]. Reducing HSV-2 infection severity (number and severity of lesions, and immune cell presence) should be expected to reduce the risk of HIV-1 infection; however, clinical studies of HSV-2 positive individuals found that the incidence of HIV-1 infection was not reduced by (val)acyclovir treatment [21, 23, 24]. The reason for this discrepancy remains unknown. Some studies have argued that the doses given in these studies may simply have not been high enough [24]. Others point to the short half-lives of these drugs, which may be insufficient to completely suppress the extremely rapid kinetics of HSV-2 replication [13]. Determining whether other antivirals such as pritelivir, which has a much longer half-life and suppresses HSV-2 replication better than (val)acyclovir, may in fact decrease HIV-1 infection rates would have important global health implications. The use of mathematical models can provide insight into HSV-2 and HIV-1 infection dynamics in patients receiving antivirals, and may help to determine correct dosage amounts.

While a considerable amount of mathematical modelling has focussed on HIV-1 or HSV-2 infections independently [7, 9, 11–13, 25–28], none have analyzed the establishment of HIV-1 coinfection in individuals with chronic HSV-2 from a mechanistic and immunological perspective. Further, mathematical models have yet to be utilized to understand how antiviral drugs may decrease the risk of HIV-1 infection in persons infected with HSV-2. Here, we apply a spatial stochastic model to describe the dynamics of a genital herpetic lesion caused by HSV-2, and quantify the risk of HIV-1 acquisition based on an individual’s current state of HSV-2 infection. Using our model, we study the effects of HSV-2 antiviral drugs on HSV-2 outbreaks and the likelihood of HIV-1 infection following exposure. We also predict the dosage of antiviral drugs needed to achieve significant reductions in the HIV-1 infection probability. Our modelling predictions are consistent with previous work and can be tested by future studies on HIV-1 acquisition in treated and untreated HSV-2 patients.

## Results

### Spatial model of HSV-2 lesion development

**Fig 1:**
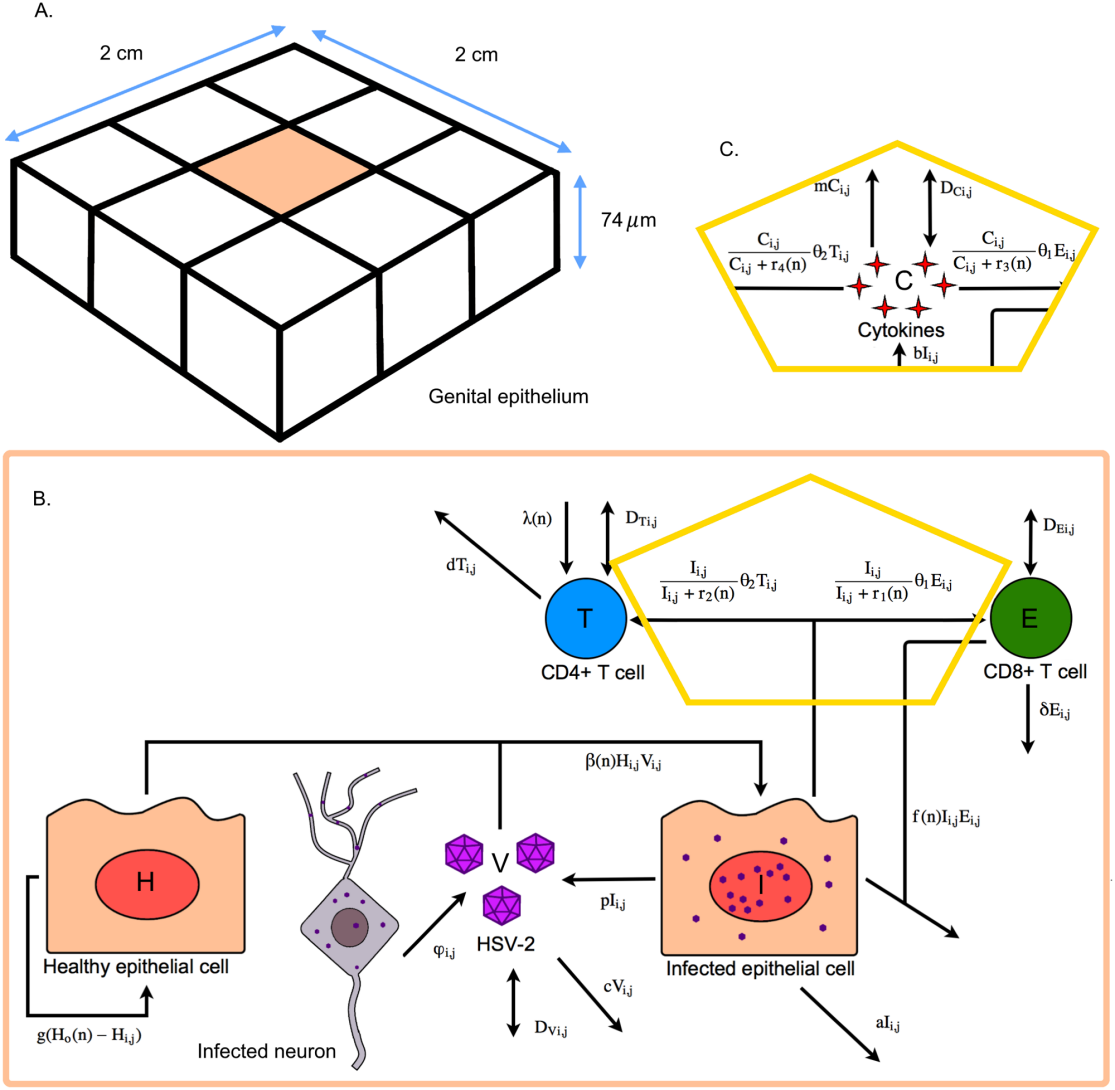
Spatial mathematical model of HSV-2 infection. (A) 2 cm × 2 cm × 74 *μ*m patch of genital epithelium that is described by the model, divided into *n* × *n* grid sites. (B) Model diagram indicating the viral, epithelial, and immune cell dynamics that occur within one grid site of the modelled epithelium. (C) Modified version of the model, including cytokine dynamics. This fragment replaces the yellow outlined region of the first model.

We begin by creating a mathematical model to describe the general dynamics of a chronic HSV-2 infection in a small region of the genital mucosa. As herpetic lesions rarely reach diameters exceeding 6mm [7], we model a 2cm × 2cm region of the mucosa. We model the tissue to a depth of 74 *μ*m, representing the average thickness of infectible epithelial tissue as measured by previous histological studies [7]. We further divide the model region into *n* × *n* equally sized cuboidal grid sites to spatially resolve lesion development. A schematic of the model is shown in Fig 1 and full details are given in the Methods.

We developed two versions of the model. In both, the important dynamics and interactions between HSV-2 free virus (V), infected and healthy epithelial cells (I and H respectively), and CD4+ and CD8+ immune cells (T and E respectively) are included. The two models differ based on assumptions around how the mucosal immune response is induced. The first, and simpler, version of the model assumes that immune cell proliferation is proportional to the number of infected epithelial cells (Fig 1B), following previous models of HSV-2 lesion dynamics [7, 9, 11–13]. The second version incorporates the effects of cytokines (C) produced by epithelial cells in response to infection (Fig 1C).

#### Simulations of HSV-2 lesion development

**Fig 2:**
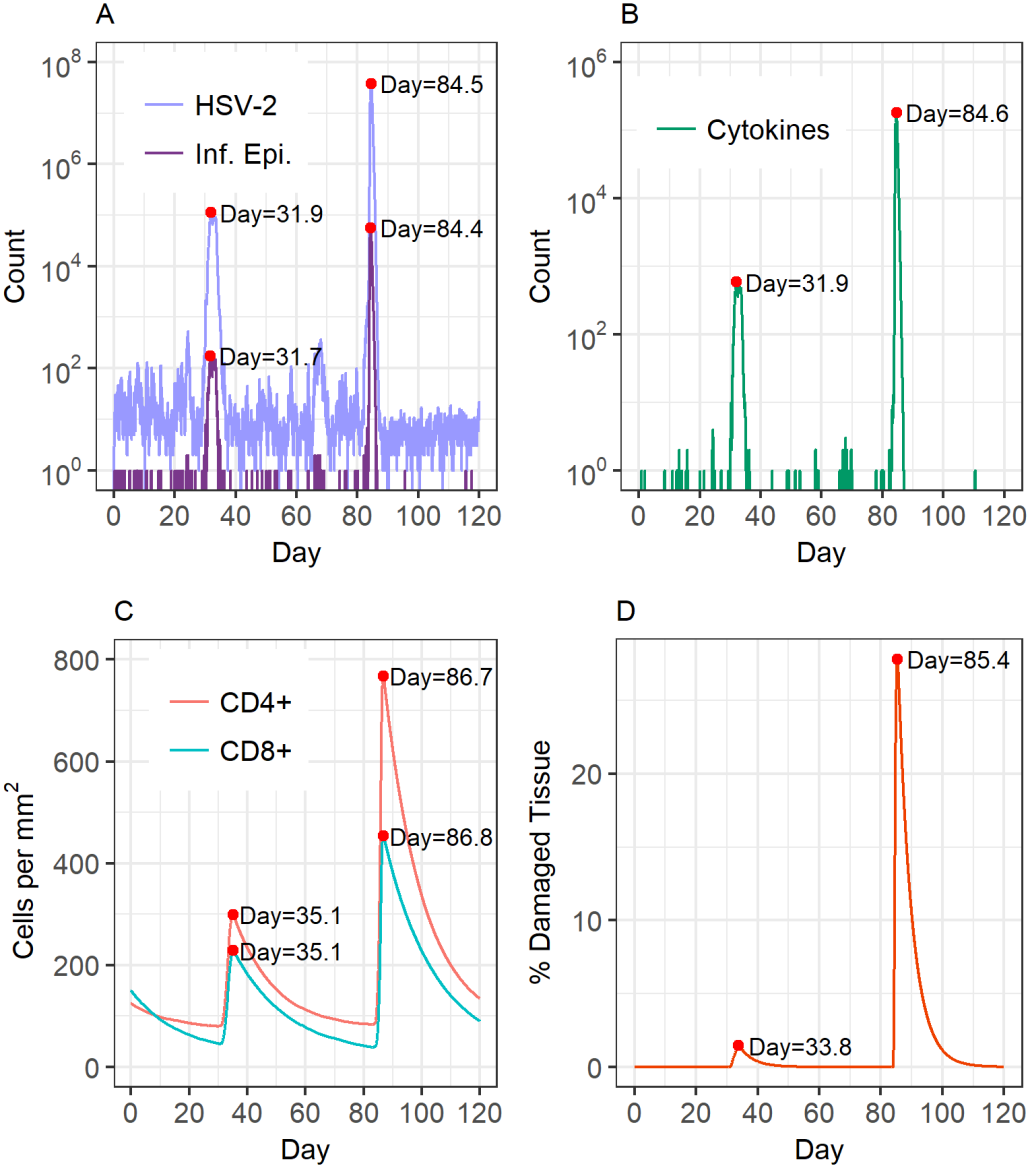
HSV-2 infection dynamics from a single model simulation. (A) Total (summed across the whole simulation region) HSV-2 and infected epithelial cell counts. (B) Cytokine concentration. (C) CD4+ and CD8+ T cell densities. (D) Percentage of the modelled region damaged due to lesion development. The time at which each tracked value peaks during a HSV-2 outbreak is indicated to highlight the delay in immune cell response and tissue damage.

**Fig 3:**
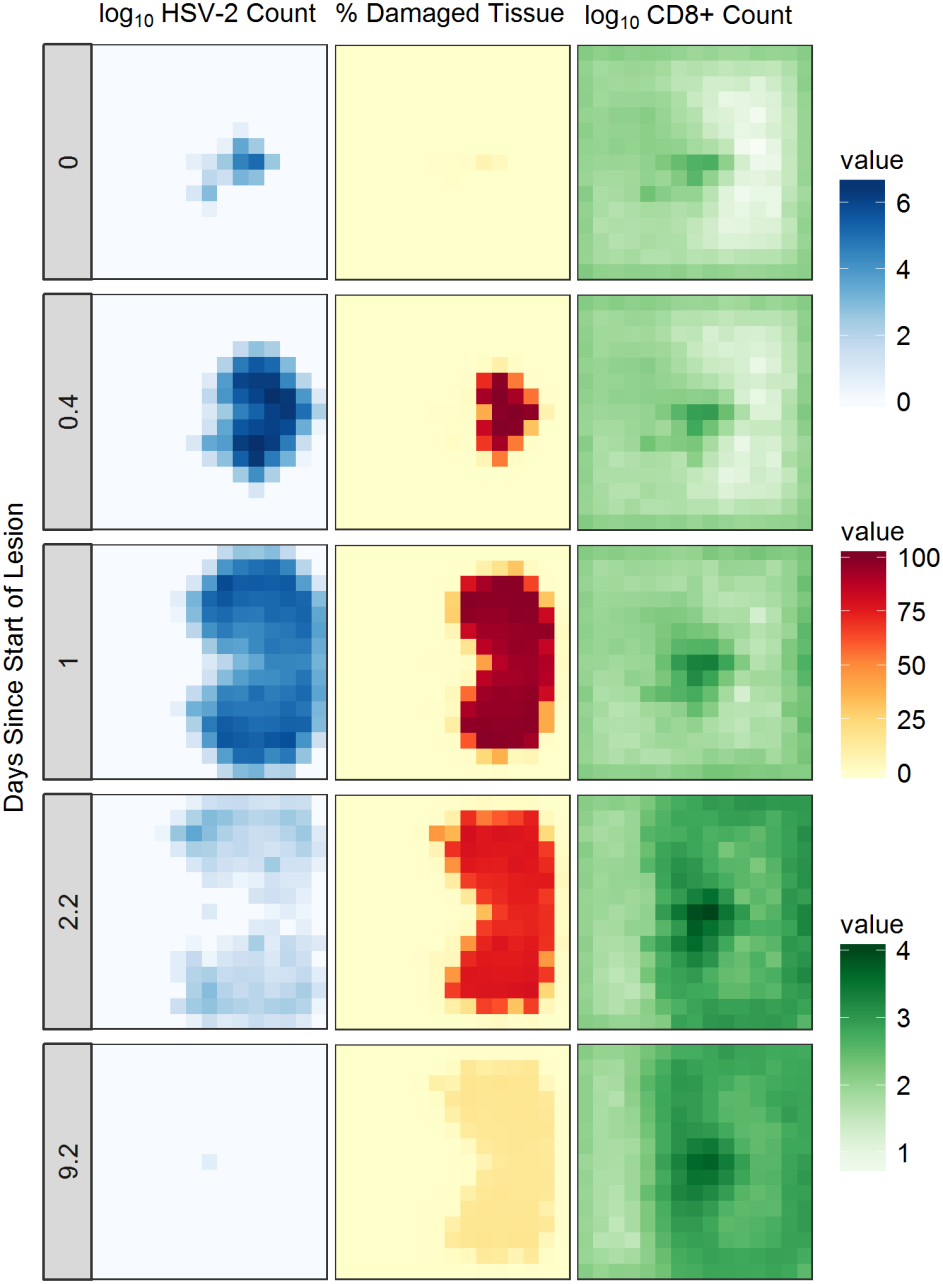
Spatial dynamics of HSV-2 infection and lesion development. Stills of HSV-2 lesion development taken from a single model simulation including the effects of cytokines. Log_10_ HSV-2 counts (left), percentages of tissue damage due to lesion development (centre), and log_10_ CD8+ immune cell counts (right) are shown over time as the lesion develops and then heals.

**Fig 4:**
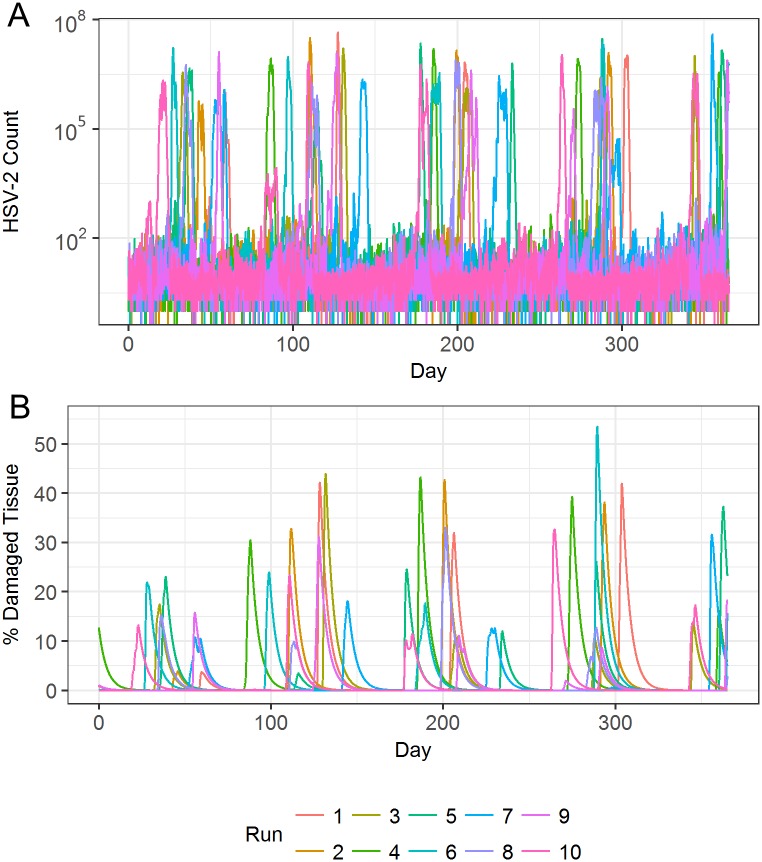
HSV-2 infection dynamics from multiple model simulations. (A) Total quantity of HSV-2 within the simulation region at all time points throughout year long simulations. (B) Total percentage of tissue damage within the simulation region at all time points throughout year long simulations. Ten individual simulations are shown.

We found that the first (no explicit cytokine) version of the model produced simulations where tissue damage spreads uncontrolled (S1 Fig). However, the second model (which explicitly models the development and spread of cytokines) allows for damaged tissue to be contained and ultimately repaired, yielding a realistic representation of HSV-2 lesion development in the genital tract. Here, we define a lesion as a spatially localized patch of damaged tissue. In order for the first model to present realistic lesion dynamics, there would need to be an unrealistically fast immune response to keep up with the spread of the virus. When cytokines are not present in our simulations, immune cells arriving at sites with many infected cells are always one step behind the virus, and cannot “catch up” with the infection. When we add fast-diffusing cytokines (which can keep up with, or surpass, the diffusion speed of the virus) as the stimulants of immune response, the immune cells are able to get ahead of the virus and protect tissue before it becomes infected. Snapshots of modelled HSV-2 infection dynamics with the inclusion of cytokines are shown in Figs 2–4. Figs 2 and 3 show the results of a single simulation. In Fig 2, total virus, cell, and cytokine counts within the simulation region are shown over time. Infection dynamics are broadly consistent with those in the literature [4, 7]. We find that peaks in HSV-2 directly correspond with peaks in infected cells and cytokines while corresponding peaks in immune cells and tissue damage are delayed. This captures the known lag in tissue response to infection. Once immune cells respond, virus is cleared quickly and tissue begins to slowly heal. Immune cell counts remain elevated long after the lesion episode, consistent with their role in suppressing the establishment of new lesions [9]. New viral peaks occur after the immune cell presence has waned, in line with an effective threshold in local immune conditions, above which new lesions are unable to form.

Fig 3 shows spatially resolved simulation data depicting the development of a single lesion over time. With the inclusion of cytokines, simulations present lesions as a connected, growing patch. The lesion grows to a biologically realistic maximum size within the simulation area and then slowly heals. The CD8+ T cell presence becomes concentrated in areas of high infection, and remains at high, and protective, concentrations even once virus has been (temporarily) cleared from the system.

Fig 4 shows results from multiple model simulations, capturing the variation in viral loads and lesion sizes seen in different simulated patients. In the absence of large lesions, simulations often show small amounts of virus existing in the system, matching the asymptomatic viral shedding that is well described in chronically infected patients [7]. Occasionally, more severe infections become established in the tissue, leading to a high viral peak and subsequently a large amount of tissue damage. At times of severe lesions, tissue damage can account for up to 50% of the 4 cm^2^ patch of tissue, with HSV-2 reaching close to 10^8^ viral copies per lesion. These large lesions are in general agreement with clinical observations [29].

Having achieved a good quantitative model of lesion development that is in line with available clinical and experimental data, we now move on to study the HIV-1 infection process in the presence of such a lesion.

### Model of HIV-1 acquisition in HSV-2 infected patients

**Fig 5:**
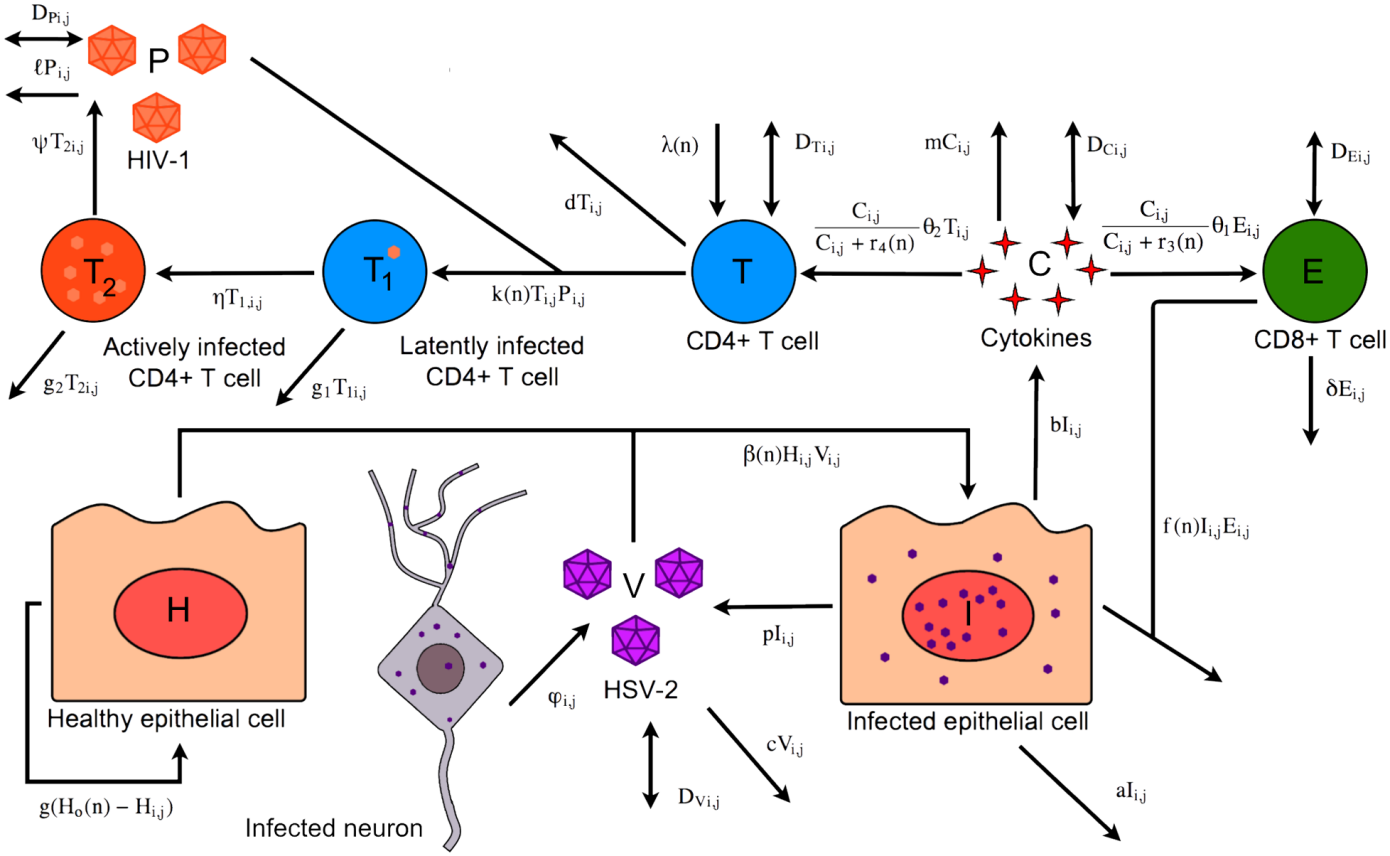
Spatial mathematical model of HIV-1 infection within HSV-2 infected tissue. Model diagram indicating the dynamics of HIV-1, HSV-2, and epithelial and immune cell dynamics that occur when patients experience co-infection or an invading HIV-1 infection.

We modified our model to examine how the characteristics of an HSV-2 infection may affect the likelihood of HIV-1 acquisition in women exposed to HIV-1. Briefly, we added cell and viral populations and interactions that capture the basic processes: infection of CD4+ T cells by HIV-1, conversion of these cells into those which actively produce HIV-1, and the production and decay of extracellular virus. Our modified model is shown schematically in Fig 5 and described in full detail in the Methods.

**Fig 6:**
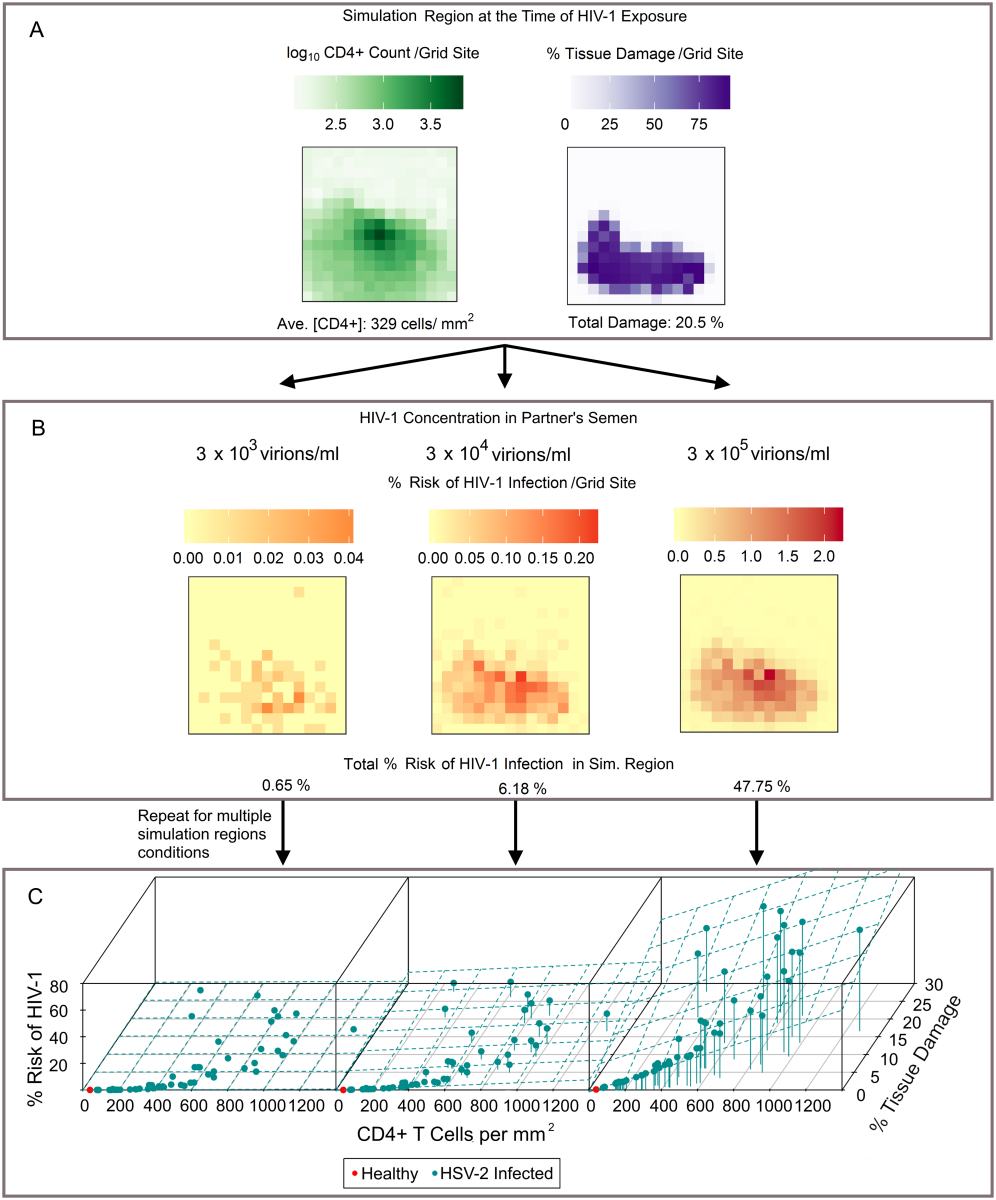
Probability of HIV-1 infection as dictated by CD4+ T cell count and damaged tissue amount. (A) Spatial depiction of the probability of HIV-1 infection within one representative simulated tissue sample. Left: CD4+ T cell counts (cells/mm^2^). Right: the amount of tissue damage in the simulation region at the time of exposure. (B) The probability of an HIV-1 infection when the simulation region in (A) is exposed to three different seminal HIV-1 concentrations during vaginal intercourse. Combining grid site-specific probabilities leads to an overall 0.65%, 6.18% risk, or 47.75% risk of HIV-1 infection establishment within the model region depending on the HIV-1 exposure level. (C) Probability of HIV-1 infection across 61 simulation tissue samples and for each seminal HIV-1 concentration. HIV-1 risk is plotted against the number of CD4+ T cells per mm^2^ and the percentage of herpetic lesion tissue damage within the simulation region at the time of HIV-1 introduction. The single red points indicate the risk of HIV-1 contraction if tissue is healthy. Linear planes of fit for each set of data points are also shown (Eqs 1, 2 and 3 from left to right, with adjusted R^2^ = 0.9702, 0.9797, and 0.9951 respectively).

HSV-2 infections are thought to increase HIV-1 infectivity in two ways: by creating an epithelial lesion that serves as a viral entry point, and by providing a simultaneous increase in the local number of CD4+ target cells. To determine how these factors affect the probability of HIV-1 infection following exposure, we chose to examine the scenario where the genital tissue of HSV-2 females is exposed to HIV-1 through sexual encounters with HIV-1 infected males. HSV-2 simulations were “paused” at different points during lesion development, creating spatially explicit simulated tissue samples (Fig 6A). We then introduced HIV-1 to each simulated tissue sample in an amount proportional to that found within the semen of HIV-1 infected males (3 × 10^3^ – 3 × 10^5^ virions/mL) and to the current amount of tissue damage within the sample. Using this initial condition, we simulated dynamics of HIV-1 infection and clearance, as described in the Methods. By running many HIV-1 simulations for each HSV-2 condition, we were able to determine the probability of HIV-1 infection given the current state of the female’s genital tissue (Fig 6B). We implicitly assumed that the dynamics of HIV-1 infection (or failure to infect) occur quickly on the timescale of the HSV-2 model. To test this assumption, we examined the time it takes for the infecting HIV-1 to either take hold, or go extinct. We found that in the vast majority of simulations, the HIV-1 dynamics were indeed very rapid (see S2 Fig), justifying our assumption.

We used this process to examine different simulated tissue samples. In particular, we focused on HIV-1 exposure to tissue that was healthy with no HSV-2 infection (1 sample), tissue exposed one week before the peak severity of a lesion (10 samples), tissue exposed at the time of peak severity (10 samples), tissue exposed during peak CD4+ T cell levels (10 samples), and tissue exposed one, two, or four weeks after the peak severity of a lesion (10 samples each). In total, this led to an analysis of 61 tissue samples. Properties of these simulated tissue samples are summarized in Table 1.

**Table 1:** Simulated tissue sample properties relevant to HIV-1 infection probability.

Simulation Initial Conditions	# of Samples	Mean CD4+ cell count/mm^2^ Across Samples	Mean % Tissue Damage Across Samples
healthy	1	40	0
1 week before peak lesion	10	82	0.001
peak lesion	10	601	15.771
peak CD4+ cell count	10	876	12.2
1 week after peak lesion	10	616	3.7
2 weeks after peak lesion	10	371	0.7
4 weeks after peak lesion	10	181	0.06

We plot the probability of HIV-1 infection for each of the 61 samples against the number of CD4+ T cells and the amount of tissue damage at the time of HIV-1 introduction as shown in Fig 6C. We do this for three possible seminal concentrations of HIV-1. By fitting linear planes to the data, the probability of HIV-1 infection can be expressed as a simple function of the CD4+ T cell concentration and the amount of tissue damage within the simulation region at the time of exposure.

When a simulation region is exposed to seminal HIV-1 concentrations of 3 × 10^3^ virions/mL, characteristic of chronic HIV-1 infection, HIV-1 infection probability within the simulation region can be expressed as

$$\begin{array}{c} \text{Prob(infection}\;\text{in}\;\text{sim}\;\text{region)}\approx 4.8\times 10^{-4}T+0.033L-0.014 \end{array}$$ (1)

where *T* represents the CD4+ T cell concentration (cells/mm^2^) and *L* represents the percentage of damaged tissue within the simulation region. The corresponding linear approximations for the probability of infection following exposure to seminal HIV-1 concentrations of 3 × 10^4^ virions/mL and 3 × 10^5^ virions/mL were found to be

$$\begin{array}{c} \text{Prob(infection}\;\text{in}\;\text{sim}\;\text{region)}\approx 5.6\times 10^{-3}T+0.29L-0.21 \end{array}$$ (2)

and

$$\begin{array}{c} \text{Prob(infection}\;\text{in}\;\text{sim}\;\text{region)}\approx 4.4\times 10^{-2}T+1.65L-1.41 \end{array}$$ (3)

respectively. Note that the numerical values in these equations are dimensional, with appropriate units so that each overall probability is dimensionless. In each case, we constrain the fit so that the function passes through the point representing the HIV-1 infection probability in healthy tissue predicted by the model (0.005%, 0.02% and 0.34% respectively). We note that in all scenarios, increases in either CD4+ T cell count or tissue damage increase the risk of HIV-1 acquisition.

A strong reason to use a spatial model when estimating the risk of HIV-1 infection in HSV-2-affected tissue is that there is likely to be a correlation between the location of tissue damage and the location of CD4+ T cells. HIV-1 is expected to penetrate into regions of greater tissue damage with higher probability, where it can encounter high CD4+ T cell concentrations, thus increasing the probability of successful infection above what might be expected if this spatial correlation is not considered. An artificial example of how the distribution of tissue damage and CD4+ T cell count within a simulation region can change the probability of HIV-1 infection is shown in S3 Fig.

To further investigate the importance of spatial effects, we estimated the risk of HIV-1 acquisition using a non-spatial version of the model. When we remove the spatial aspect, we effectively assume that tissue damage and CD4+ T cell counts are uniform across the simulation region, removing possible hot spots for infection. To perform these simulations, we introduced HIV-1 into the same simulated tissue samples as examined above, but after pooling all sub-volume data. We then fit the risk of HIV-1 infection as a function of CD4+ cell count and the percentage of tissue damage, to produce equations similar to those given above (Eqs 1–3). In the first two scenarios, corresponding to exposure to semen concentrations of 3 × 10^3^ and 3 × 10^4^ virions/mL, the fits did not change substantially (changes in linear fit coefficients of less than 20%). However, in the third scenario, (semen HIV-1 concentration of 3 × 10^5^ virions/mL), the loss of spatial effects caused a large decrease in the estimate of HIV-1 risk. This was due to the (imperfect) spatial correlation between the location of CD4+ T cells and damaged tissue, driven by the delay in CD4+ T cell response as noted in Fig 2.

**Fig 7:**
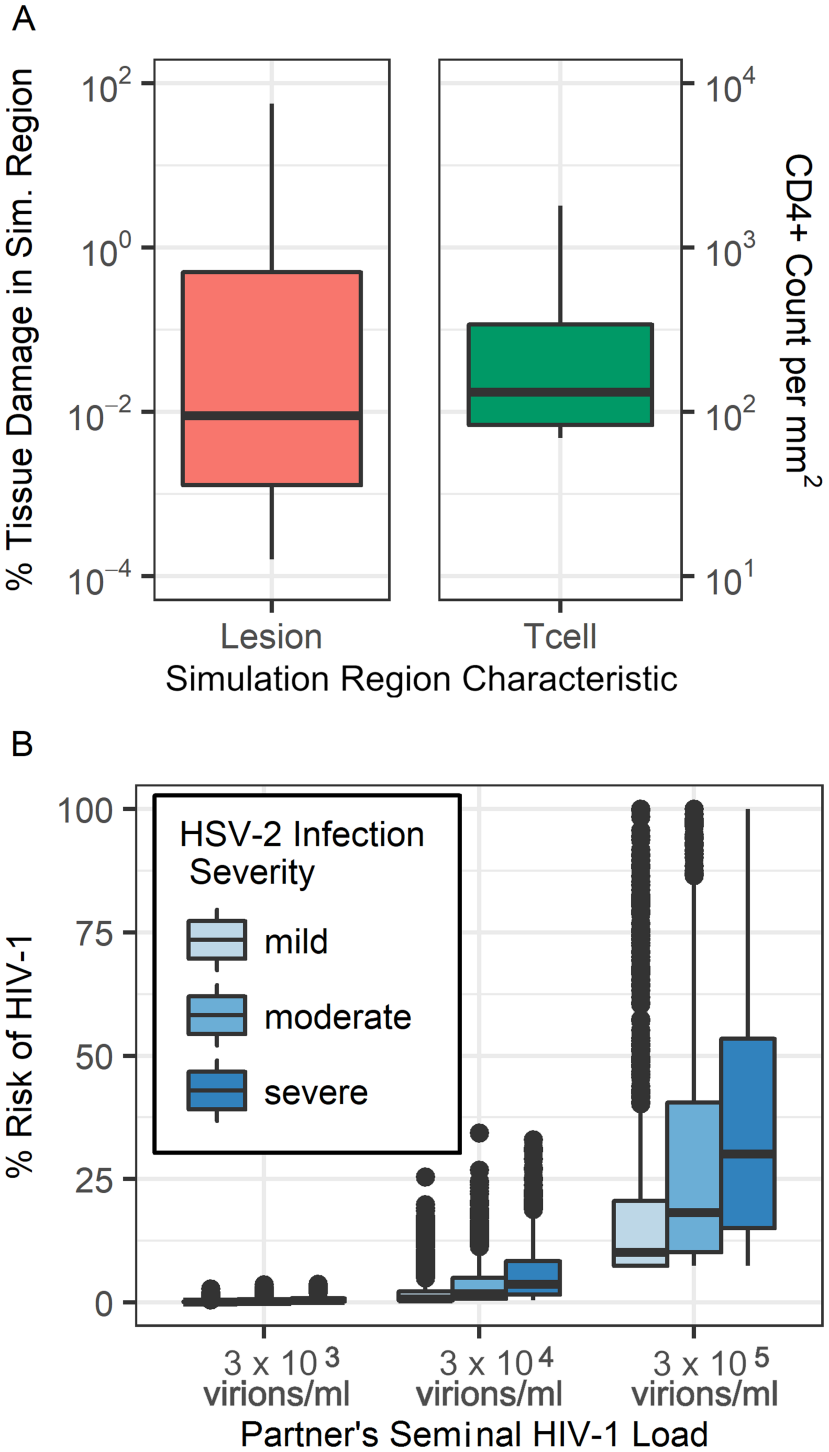
Risk of HIV-1 infection for the entire genital tract. (A) Distribution of CD4+ T cell counts and the amount of tissue damage seen across simulations of the model region. (B) A female’s risk of HIV-1 contraction per sexual act with an HIV-1 infected male partner. Risk is stratified by both the severity of HSV-2 infection in the female and the HIV-1 concentration in the semen of the male partner.

As the results so far only consider a single (4cm^2^) patch of genital epithelium, we next calculated the risk of HIV-1 over the entire genital tract. The female genital tract has a surface area of approximately 88 cm^2^ [30], corresponding to 22 of our simulation regions. Results taken from 50 simulations each of length 12 months were combined to obtain the distributions of CD4+ counts and levels of tissue damage at sites surrounding HSV-2 infected neurons (Fig 7A). We sampled from these distributions, to create a set of genital tract profiles (each composed of 22 simulation regions) for HSV-2 infected patients, and then calculated the probability of infection at each site in the genital tract profile using Eqs 1–3. These risks were then combined to calculate the risk of HIV-1 infection per sexual act when exposed to different per-ejaculate HIV-1 viral loads. As expected, high seminal HIV-1 viral loads lead to higher probability of infection. Since an HSV-2 infected individual will have some regions of healthy tissue and others with infected neurons releasing HSV-2, not all 22 regions were considered lesioned, but instead some were assumed to simply possess the characteristics of healthy tissue. Similarly, some individuals will have more infected regions than others, thus we varied the number of simulation regions assumed to have neurons dripping HSV-2 into the system. We defined three severities of HSV-2 infection as follows: *Mild*, *moderate* and *severe* infections were respectively defined to have 1, 2, and 3 simulation regions (out of the 22 regions) where HSV-2 is actively being introduced into the system. The severity of HSV-2 infection is found to significantly alter the risk of HIV-1 infection after exposure (Fig 7B). When weighing how tissue damage and CD4+ cell counts each contribute to this risk, CD4+ cells are the main contributor. Lesions are sporadic and their durations are relatively short; this means they only contribute to HIV-1 risk a fraction of the time. CD4+ T cell counts remain elevated for much longer, indicating that for the majority of time the density of CD4+ T cells in the mucosa is an important driver of HIV-1 acquisition.

### Effects of HSV-2 treatment on lesion severity and risk of HIV-1 acquisition

**Fig 8:**
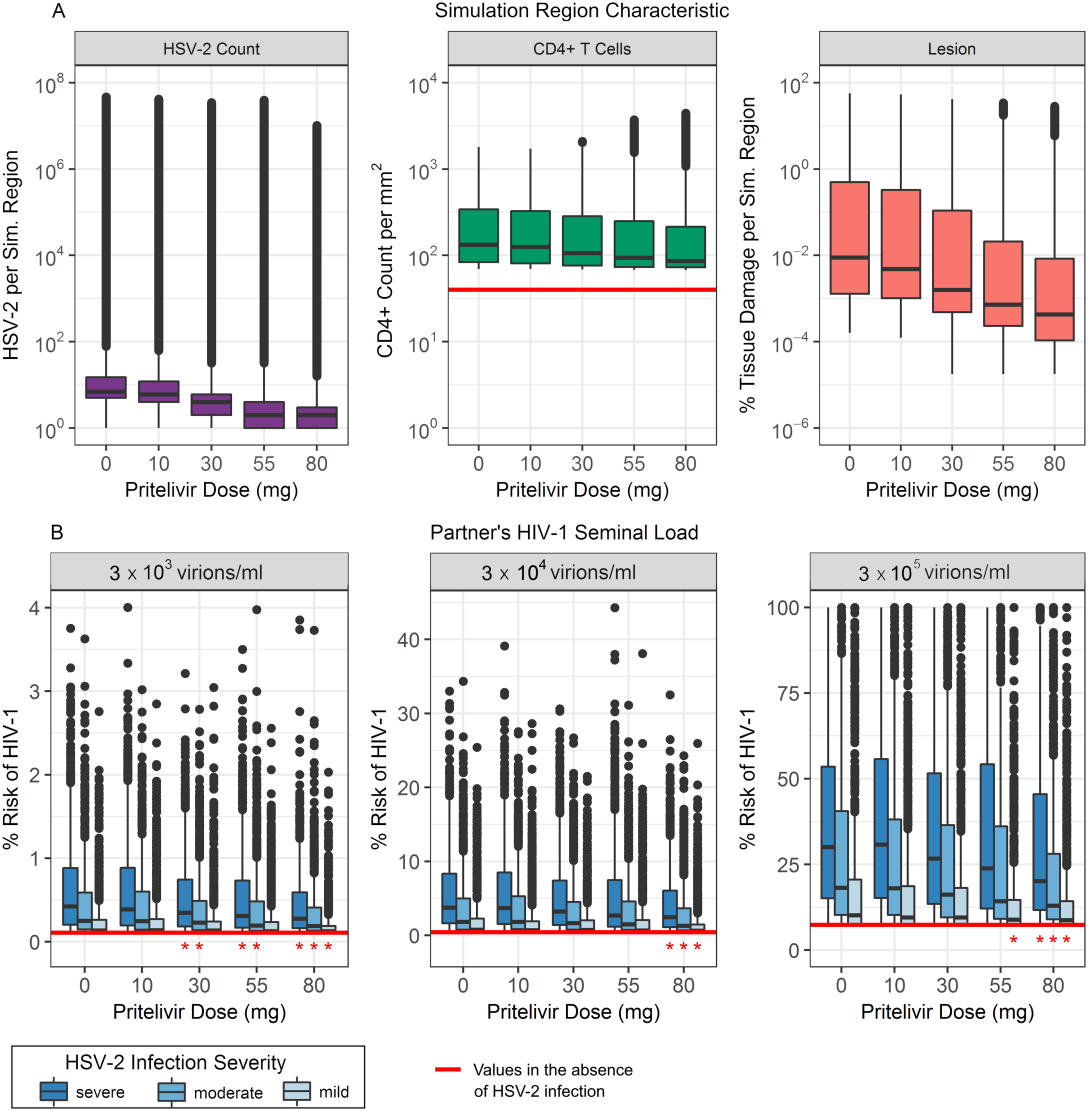
The effect of antivirals on HSV-2 lesions and the risk of HIV-1 infection. 50 model simulations, each lasting 12 months, were performed for each of 5 different daily doses of pritelivir. (A) Distributions of HSV-2 count, CD4+ T cell count per mm^2^, and the percentage of tissue damage within one simulation region. (B) Risk of HIV-1 infection in females, stratified by the severity of the HSV-2 infection and the concentration of HIV-1 in the semen at the time of exposure. Horizontal red lines indicate levels observed in the absence of HSV-2 infection. For each HSV-2 infection severity and each HIV-1 seminal load, doses of pritelivir that significantly decrease the risk of HIV-1 contraction below those of simulated untreated patients are indicated by red stars (p<0.01).

Pritelivir is a recently developed antiviral for HSV-2 treatment. It acts as a viral helicase-primase inhibitor, preventing viral replication, and is currently in clinical trials. We examined how doses of 10, 30, 55, and 80 mg/day would be predicted to affect the characteristics of HSV-2 infection and the corresponding risk of contracting HIV-1. These doses all fall within the range of doses from recent drug trials [31]. In Fig 8A–8C we show distributions of HSV-2, CD4+ T cell, and total tissue damage within one simulation region, obtained from fifty one-year-long simulations at each drug dose. As the pritelivir dose increases, the number of HSV-2 virions and amount of tissue damage associated with a lesion decreases accordingly. The median CD4+ T cell count, however, is relatively unaffected by these particular doses of pritelivir, remaining at more than double the amount seen in healthy tissue even at the highest examined pritelivir dose [4]. As the number of tissue-resident CD4+ T cells is thought to affect the risk of HIV-1 acquisition, this result indicates a deficiency of HSV-2 antiviral strategies in preventing HIV-1 infection.

To further investigate this hypothesis, we used our simulated data on CD4+ T cell densities and epithelial tissue damage to calculate the overall risk of HIV-1 infection when the entire female genital tract is exposed to HIV-1 in semen (Fig 8). We again examined scenarios where patients have different severities of HSV-2 infection determined by the number of sites in the genital epithelium (1, 2, or 3) where HSV-2 is actively being introduced by infected neurons.

For a female with completely healthy tissue, the per-sexual-act probability of HIV-1 infection when exposed to per-ejaculate HIV-1 concentrations of 3 × 10^3^, 3 × 10^4^, or 3 × 10^5^ virions/mL was calculated to be 0.11%, 0.44% and 7.41% respectively. These percentages are indicated by the red horizontal lines in Fig 8B and match values reported in the literature [5, 32–36]. In patients not receiving antivirals, studies have recorded HIV-1 infection risk to be 2-3 times higher in asymptomatic HSV-2 infection, compared to healthy patients, and 7 times higher if lesions are present [2, 37]. Our simulations were in general agreement with these observations. The smallest median increase in HIV-1 risk due to HSV-2 infection was 1.2 fold (1.0-9.1 fold from 5th to 95th percentile), seen in simulations where HSV-2 infection was considered mild and tissue was exposed to the lowest HIV-1 semen concentration. The highest median increase in HIV-1 risk due to HSV-2 infection was 8.6 fold (1.4-39.6 fold), seen in simulations where HSV-2 infection was considered severe and tissue was exposed to an intermediate HIV-1 semen concentration.

When simulations included pritelivir treatment, the risk of HIV-1 infection was significantly decreased (p<0.01) at some pritelivir doses (Fig 8B). In some cases, 30 mg/day was enough to cause a significant decrease in HIV-1 infection risk, and pritelivir doses of 80 mg/day showed significant decrease in risk for all scenarios examined. Despite this, none of the pritelivir doses were able to return the risk of HIV-1 infection to the baseline values seen in healthy tissue. Even at the highest dose of pritelivir, the median risk of HIV-1 contraction at best remained 1.2-fold higher (1.0-6.1 fold from 5th to 95th percentile) than that seen in healthy patient simulations (occurring when HSV-2 infection was mild and HIV-1 semen concentrations were low) and at worst remained a median of 5.6-fold higher (1.4-34.2 fold) than that seen in healthy patient simulations (occurring when HSV-2 infection was severe and HIV-1 semen concentrations were moderate).

## Discussion

By developing a spatial stochastic model to describe the dynamics of chronic HSV-2 infections in the genital mucosa, we determined the characteristics of infection essential for describing the growth and control of HSV-2 lesions, and how these traits can be directly used to predict the risk of HIV-1 infection.

We found that immune cells alone were not able to control the spatial spread of lesions. This was due to their slow mobility within the tissue and their inability to keep up with the spread of infection. As we included in our preferred model, rapidly diffusing cytokines were needed to stimulate the recruitment and proliferation of immune cells to control the spread of infection. Other responses to cytokines, such as the antiviral state uninfected cells can enter, may also play an important role in controlling lesion growth, as suggested in previous models of viral dynamics [38]. We did not include these effects in the present work, but they may prove important. Future work may include examining the roles of each of these factors in controlling simulated lesion development.

Using our model, we also determined how the risk of HIV-1 infection in women through vaginal intercourse depends on the extent of genital HSV-2 infection. While it has long been accepted that HSV-2 infection increases the risk of HIV-1 acquisition, the relationship has not been quantified in detail. We have argued that the risk of HIV-1 infection is controlled by the local concentration of CD4+ T cells and the degree of damage caused by HSV-2 within the genital tissue. The severity of HSV-2 infection is often measured by the viral load in genital swabs; however, our model indicates that HSV-2 viral load is only weakly correlated with HIV-1 risk. When HSV-2 viral loads peak due to a new lesion, there is a delay before tissue damage and CD4+ T cell counts peak due to the nature of the tissue’s response to infection. This means that HSV-2 viral load is not an effective metric of HIV-1 susceptibility.

As HSV-2 antivirals decrease the severity of HSV-2 infection, we wanted to quantify how these effects might decrease the risk of HIV-1 acquisition. With a particular focus on the newly developed HSV-2 antiviral drug pritelivir, we showed that certain doses may decrease the risk factors linked with HIV-1 infection susceptibility, namely CD4+ T cell count and HSV-2 lesion severity. These decreases led to a significant overall decrease in HIV-1 acquisition risk, as predicted by our simulations. While such a decrease in risk from HSV-2 antiviral drugs has been predicted, clinical studies have failed to detect the effect [23, 24].

There are several possible reasons for this disconnect between theory and data. In clinical studies observing the effect of HSV-2 antivirals on HIV-1 infection probability, many participants were co-infected with other STIs and this effect was not controlled for [23, 24]. Non-ulcerative STIs can increase CD4+ T cell levels to twice those seen in healthy tissue [39]. Our results indicate that CD4+ T cell counts in the genital mucosa are the greatest determinant of HIV-1 infection risk, meaning higher CD4+ T cell counts due to other STIs may have kept CD4+ T cell counts too high for significant reductions in HIV-1 infection risk.

Another reason for this disconnect may be that clinical studies aimed at detecting the effects of HSV-2 antivirals on HIV-1 infection used acyclovir as their study drug. Acyclovir has rapid pharmacokinetics with a short half-life of 3-4 hours [23, 24]. Previous mathematical models of HSV-2 infection that included the effects of antiviral drug decay show that when drug concentrations reach sub-therapeutic levels, rapid HSV-2 breakouts can occur, preventing full control of the infection [13]. Lack of HIV-1 risk reduction observed in these acyclovir studies may thus be explained by sub-therapeutic drug levels. Pritelivir has a half-life estimated to be approximately 80 hours [13] which should reduce the effects of drug decay and allow for better control of HSV-2 infection breakouts and HIV-1 infection probability.

In future work, it may be also interesting to examine how the effects of HIV-1 antibodies or HIV pre-exposure prophylaxis (PrEP) could change the risk of HIV-1 contraction in HSV-2 patients. A recent study has examined how antibody presence and efficacy may affect the transmission of HIV-1 to healthy individuals [35]. Calibrating this model to include the effects of HSV-2 infection may provide interesting insights as further preventative measures against HIV-1 are developed.

As with all mathematical modelling work, our study has some limitations. A primary limitation is related to the role of CD4+ T cells in controlling HSV-2. We incorporated CD4+ T cells in our model as an essential part of studying HIV-1 infection. However, the direct effect of CD4+ T cells on the control of HSV-2 in the mucosa remains unclear. As more experimental information on the role of CD4+ T cells becomes available, it will be important to revise and improve our model. New developments also may support further mathematical modelling of the synergy between HSV-2 and HIV-1 from the immunological perspective.

In conclusion, our work provides insight to herpes lesion development in the genital mucosa and forms a logical framework for studying the synergy between HSV-2 and HIV-1 infections. Our results support the use of pritelivir or other HSV-2 antivirals as an effective means of preventing HIV-1 infection in patients infected with HSV-2, and highlight the essential mechanisms by which pritelivir reduces the risk of HIV-1. While clinical studies have been performed to examine the effects of pritelivir on HSV-2 infection alone, none have yet examined its effect on the risk of HIV-1 infection. Furthermore, the modelling framework laid out here to examine the spatial development of lesions in the genital epithelium could potentially be expanded to mathematical studies of other skin abnormalities, building on experimental knowledge of tissue-resident immune cells and their interactions with invading pathogens or (pre-)cancerous cells.

## Materials and methods

### HSV-2 model design

We focus on a 2cm × 2cm × 74*μ*m volume of genital epithelium. This is modelled as a single-layer *n* × *n* array of cuboidal subvolumes. The assumptions of the model (described in turn below and summarized in Fig 1) are used to form a chemical master equation that describes the dynamics of all possible reactions in the system. Following Gillespie’s direct simulation algorithm, modified to allow for spatially separated sites, the model is executed to produce one simulation [40, 41]. We assume that reactions only occur between cells, virions, and cytokines that exist within the same grid site, while motion of an object from one location to another is modelled as a distinct reaction (with fixed rate).

In the absence of infection, suppose each subvolume contains *H*_0_(*n*) healthy epithelial cells. Taking the diameter of a healthy epithelial cell to be ∼17 *μ*m and assuming a roughly cuboidal cell shape gives approximately 6 × 10^6^ cells inside the entire model region [7]. We therefore set *H*_0_(*n*) = 6 × 10^6^/*n*^2^. Defining the actual number of epithelial cells in a given subvolume (indexed by *i* and *j*) as *H*_*i*, *j*_, we model tissue repair as a simple exponential return to the healthy state with rate *g*. This is described as the state-dependent reaction

$$\begin{array}{c} H_{i,j}\to H_{i,j}+1\;\;\text{with}\;\text{rate}\;g\left(H_{0}\left(n\right)-H_{i,j}\right). \end{array}$$ (4)

HSV-2 infections are initiated by the release of virus from neurons innervating the genital mucosa, which occurs at a constant rate *ϕ*_*i*, *j*_. The model region is assumed to be centered around an HSV-2 infected neuron meaning *ϕ*_*i*, *j*_ = 0 for all grid sites except for the center site (*ic*, *jc*). Healthy epithelial cells become infected by HSV-2 virus following the law of mass action at a rate proportional to *β*(*n*), and new virus is produced by these infected cells at a per-capita rate *p*. Free HSV-2 virus decays at a per-capita rate *c*. These events are described by the following set of reactions:

$$\begin{array}{ccc} V_{ic,jc}\to V_{ic,jc}+1\; & \;\text{with}\;\text{rate}\; & \phi_{ic,jc} \end{array}$$ (5)

$$\begin{array}{ccc} \{V_{i,j}\to V_{i,j}-1,\;H_{i,j}\to H_{i,j}-1,\;I_{i,j}\to I_{i,j}+1\}\; & \;\text{with}\;\text{rate}\; & \beta \left(n\right)H_{i,j}V_{i,j} \end{array}$$ (6)

$$\begin{array}{ccc} V_{i,j}\to V_{i,j}+1\; & \;\text{with}\;\text{rate}\; & pI_{i,j} \end{array}$$ (7)

$$\begin{array}{ccc} V_{i,j}\to V_{i,j}-1\; & \;\text{with}\;\text{rate}\; & cV_{i,j} \end{array}$$ (8)

Modelled CD8+ effector T cells (*E*_*i*, *j*_) are taken to be HSV-2 specific and interact and kill infected epithelial cells at a rate *f*(*n*), again following the law of mass action. Infected cells also die at a per-capita rate *a* as they succumb to the infection. CD8+ T cells exit the system at a small rate *δ* in keeping with the long period these cells remain at and protect previous sites of infection [4, 14]. In the infection-free state, a population of CD4+ T cells remain at the site at a per-site equilibrium number of λ(*n*)/*d*. While CD4+ T cells have not been included in previous models of HSV-2 infection [7, 9, 11–13], we include their dynamics due to their importance in the establishment of HIV-1 infections and in order to get a better representation of the full dynamics occurring at herpes lesion sites. These events are described by the following set of reactions:

$$\begin{array}{ccc} I_{i,j}\to I_{i,j}-1\; & \;\text{with}\;\text{rate}\; & \left(f\left(n\right)E_{i,j}+a\right)I_{i,j} \end{array}$$ (9)

$$\begin{array}{ccc} E_{i,j}\to E_{i,j}-1\; & \;\text{with}\;\text{rate}\; & \delta E_{i,j} \end{array}$$ (10)

$$\begin{array}{ccc} T_{i,j}\to T_{i,j}+1\; & \;\text{with}\;\text{rate}\; & \lambda (n) \end{array}$$ (11)

$$\begin{array}{ccc} T_{i,j}\to T_{i,j}-1\; & \;\text{with}\;\text{rate}\; & dT_{i,j} \end{array}$$ (12)

The presence of infection stimulates the proliferation of CD8+ T cells and CD4+ T cells in the genital mucosa. We analyze two descriptions of this stimulation. Letting *X*_1_ and *X*_2_ represent the per-capita production rate of CD8+ T cells and CD4+ T cells in response to infection respectively, these descriptions can be written as

- Model I
  $$\begin{array}{cc} X_{1} & =\frac{I_{i,j}}{I_{i,j}+r_{1}\left(n\right)}\theta_{1}, \end{array}$$ (13)
  $$\begin{array}{cc} X_{2} & =\frac{I_{i,j}}{I_{i,j}+r_{2}\left(n\right)}\theta_{2}. \end{array}$$ (14)
- Model II
  $$\begin{array}{cc} X_{1} & =\frac{C_{i,j}}{I_{i,j}+r_{3}\left(n\right)}\theta_{1}, \end{array}$$ (15)
  $$\begin{array}{cc} X_{2} & =\frac{C_{i,j}}{I_{i,j}+r_{4}\left(n\right)}\theta_{2}. \end{array}$$ (16)

In Model I, immune cell proliferation is dependent on the number of infected cells (*I*_*i*, *j*_) while in Model II immune cell proliferation is dependent on cytokine concentrations (*C*_*i*, *j*_) produced by infected cells. In both cases, immune cell proliferation saturates to a maximum per-capita proliferation rate (*θ*_1_ and *θ*_2_ for CD8+ and CD4+ T cells respectively) as the number of infected cells or cytokines increases. *r*_*i*_(*n*) determines when proliferation reaches half its maximum rate where *i* ∈ {1, 2, 3, 4}. These proliferation rates are then encoded as reaction rates for the reactions *E*_*i*, *j*_ → *E*_*i*, *j*_ + 1 and *T*_*i*, *j*_ → *T*_*i*, *j*_ + 1 respectively.

With cytokines included in Model II, we must also include their dynamics. Cytokines are assumed to be produced at a rate *b*, dependent on the number of infected epithelial cells in the same grid site, and decay from the system at a per-capita rate *m*. This corresponds to the following reactions:

$$\begin{array}{ccc} C_{i,j}\to C_{i,j}+1\; & \;\text{with}\;\text{rate}\; & bI_{i,j} \end{array}$$ (17)

$$\begin{array}{ccc} C_{i,j}\to C_{i,j}-1\; & \;\text{with}\;\text{rate}\; & mC_{i,j} \end{array}$$ (18)

We now specify the model of mobility. Since immune cells actively move around the epithelium in search of infection, and viruses and cytokines can passively diffuse through their environment, we allow HSV-2 virus, cytokines, CD4+ T cells, and CD8+ T cells to move horizontally through the tissue into neighbouring grid sites. We define the rates of motion for a specific diffusing body *N* ∈ {*E*, *T*, *V*, *C*} at grid site (*i*, *j*), where *i*, *j* ∈ {1, 2, …, *n*}, as

$$\begin{array}{cc} D_{N_{i,j}} & =\omega_{N}\frac{N_{i,j-1}+N_{i-1,j}-4N_{i,j}+N_{i+1,j}+N_{i,j+1}}{h^{2}}. \end{array}$$ (19)

Here, *ω*_*N*_ represents the motion coefficient specific to the moving body and *h*^2^ represents the horizontal cross sectional area of each grid site. These motion rates are converted into reaction rates and considered alongside all other reactions in the Gillespie simulation scheme.

At the boundaries of the simulation region, virus, cytokines, CD8+ T cells, and CD4+ T cells can exit the model region. However, as immune cells are also present in surrounding tissue, we suppose that on average there is a constant flux of CD8+ and CD4+ T cells entering the model region through its boundaries. We take *E*_*ave*_ and *T*_*ave*_ to represent the specific CD8+ and CD4+ T cell numbers expected to exist in chronic HSV-2 infected tissue, per simulation region. Then *E*_*ave*_/*n*^2^ and *T*_*ave*_/*n*^2^ represent the average CD8+ and CD4+ T cell concentrations per grid site on an *n* × *n* grid. Influx of CD8+ T cells and CD4+ T cells into boundary grid sites therefore occurs at rates *ω*_*E*_*E*_*ave*_/*n*^2^ and *ω*_*T*_*T*_*ave*_/*n*^2^ respectively. Virus and cytokines are not allowed to enter from the boundary but can exit outwards, reflecting our assumption that no other lesions are in close proximity to the modelled patch.

In Model II, we suppose that immune cells preferentially migrate up cytokine gradients [16]. To reflect this assumption, we make the direction of immune cell motion dependent on the quantity of cytokine present in neighbouring grid. The probability of immune cell motion into each of the neighbouring regions is given by

$$\text{Prob}\left(\text{motion}\;\text{into}\;(\text{i},\text{j})\right)=\frac{C_{i,j}}{C_{tot}},$$ (20)

with (*i*, *j*) taking on the indices of the four surrounding grid sites and *C*_*tot*_ being the total quantity of cytokines in these four sites. When cytokines are not present, immune cells diffuse into their four neighbouring sites with equal probability.

### Calculating tissue damage due to lesion development

We evaluate the effective tissue damage at site (*i*, *j*) as the fraction of epithelial cells removed from a particular subregion, relative to healthy uninfected tissue. This value is defined as

$$\begin{array}{c} L_{i,j}=\frac{H_{0}\left(n\right)-H_{i,j}-I_{i,j}}{H_{0}\left(n\right)}, \end{array}$$ (21)

This formula can also be summed to find the fraction of tissue damage within the entire simulation region:

$$\begin{array}{c} L_{\text{tot}}=\frac{H_{0}\left(1\right)-\sum_{i}\sum_{j}H_{i,j}-\sum_{i}\sum_{j}I_{i,j}}{H_{0}\left(1\right)}. \end{array}$$ (22)

This measure of tissue damage is used to assess the probability of HIV ingress to the simulation region, as described below.

### HIV-1 infection model

A schematic summary of the model extended to study HIV-1 infection dynamics is shown in Fig 5. HIV-1 (*P*_*i*, *j*_) propagates by infecting CD4+ T cells. This is assumed to occur through mass action at a rate *k*(*n*). Once a CD4+ cell becomes infected, it moves into the *T*_1_ class, representing latently infected cells known to be in the eclipse (not yet producing HIV) phase. These cells mature into actively infected cells (*T*_2_) at a per-capita rate *η*. Actively infected CD4+ cells produce HIV-1 virus at a per-capita rate *ψ*; this virus decays at per-capita rate *ℓ*. *T*_1_ and *T*_2_ cells may be cleared from the system at a per-capita rates *g*_1_ and *g*_2_ respectively. The model includes no specific immune response against HIV-1-infected CD4+ cells as we assume no immunity against HIV-1 infection yet exists at the earliest stage of infection. These assumptions regarding HIV infection are encoded as reactions as follows:

$$\begin{array}{ccc} \left\{T_{i,j}\to T_{i,j}-1,P_{i,j}\to P_{i,j}-1,T_{1_{i,j}}\to T_{1_{i,j}}+1\right\} & \;\text{with}\;\text{rate}\; & k\left(n\right)T_{i,j}P_{i,j} \end{array}$$ (23)

$$\begin{array}{ccc} \left\{T_{1_{i,j}}\to T_{1_{i,j}}-1,\;T_{2_{i,j}}\to T_{2_{i,j}}+1\right\} & \;\text{with}\;\text{rate}\; & \eta T_{1_{i,j}} \end{array}$$ (24)

$$\begin{array}{ccc} T_{1_{i,j}}\to T_{1_{i,j}}-1 & \;\text{with}\;\text{rate}\; & g_{1}T_{1_{i,j}} \end{array}$$ (25)

$$\begin{array}{ccc} P_{i,j}\to P_{i,j}+1\; & \;\text{with}\;\text{rate}\; & \psi T_{2_{i,j}} \end{array}$$ (26)

$$\begin{array}{ccc} P_{i,j}\to P_{i,j}-1\; & \;\text{with}\;\text{rate}\; & \ell P_{i,j} \end{array}$$ (27)

$$\begin{array}{ccc} T_{2_{i,j}}\to T_{2_{i,j}}-1 & \;\text{with}\;\text{rate}\; & g_{2}T_{2_{i,j}} \end{array}$$ (28)

HIV-1 motion is assumed to occur at rate $D_{P_{i,j}}$ with diffusion coefficient *ω*_*P*_ between neighbouring subvolumes (Eq 19 with *N* = *P*).

### Determining HIV-1 infection probability

To represent exposure to the HIV-1 virus, simulations were stopped at various points and HIV-1 was introduced into the system. To minimize computational time but still gather the data necessary to calculate HIV-1 infection probability, we tracked only the dynamics related to HIV-1 infection establishment once HIV-1 virus was introduced into the simulation region. By making this simplification, we assume that the changes in HSV-2 infection dynamics have a minimal effect on CD4+ T cell count during the small time window during which HIV-1 infection establishment occurs. This assumption was examined thoroughly by tracking the length of time it took for the infection process to reach completion (viral extinction, or the establishment of eight HIV-1 infected cells). See S2 Fig.

We chose to model the scenario where HSV-2 infected females are exposed to HIV-1 during vaginal sex with HIV-1 infected male partners. Recent *ex-vivo* experiments indicate that 0.24% of HIV-1 virions in semen penetrate the female’s genital mucosa [30]. We examined exposure to various seminal HIV-1 concentrations representative of chronic (3.0 × 10^3^ HIV-1 virions/mL), moderate (3.0 × 10^4^ HIV-1 virions/mL), and acute (3.0 × 10^5^ HIV-1 virions/mL) infections [30, 35]. Assuming that the average semen volume per ejacuate is 3 mL [36], and the vaginal surface area is approximately 88 cm^2^ [30], approximately 1, 10, or 100 HIV-1 viruses would enter our simulation region of 4 cm^2^ after a sexual encounter with a chronically, moderately, or acutely HIV-1 infected partner (respectively) when the female’s genital tissue is healthy. Carias *et al*. also found that 10 times as many viruses were able to penetrate the epithelium if weak cell junctions were present compared to tissue without weak cell junctions [30]. Assuming the effects of tissue damage are similar, we used this estimate as a conservative representation of the increased viral entry at the site of lesions. We suppose that if no lesion is present in the model region (*L*_*i*, *j*_ = 0 ∀(*i*, *j*)), then 1, 10, or 100 HIV-1 virions per simulation region enter at the time of exposure depending on the viral load in semen. These counts increase linearly with tissue damage until 10 times more virus enters the tissue if all tissue in the simulation region is damaged (*L*_*i*, *j*_ = 1 ∀(*i*, *j*)). We chose a linear relationship to minimize the complexity of the model as the exact relationship between tissue damage and virion entry remains unknown. The entry points of the virus are also made dependent on site-specific tissue damage with the virus being randomly distributed among the grid sites following a multinomial distribution with the probability of a virus being distributed to site (*i*, *j*) given by the fraction of tissue damage found at that site.

Once HIV-1 virions have been placed within the simulation region, we use Gillespie’s algorithm to calculate the infection probability for each subvolume in our computational domain. The simulation is stopped once the infection has gone extinct or propagated enough to imply infection establishment. Following previous work of Pearson *et al*., we assume that infection is effectively established once the simulation region has at least 8 infected cells [28]. Simulations for each subvolume were repeated 10,000 times. From these simulations we define the probability of HIV-1 infection as the fraction of successful infections. Finally, after running simulations for each subvolume, we combine the probabilities of infection to achieve an overall probability of HIV-1 infection for the entire simulation region:

$$\text{Prob}(\text{infection}\;\text{in}\;\text{sim}.\;\text{region})=1-\underset{i}{\prod }\underset{j}{\prod }\left[1-\text{Prob}\left(\text{infection}\;\text{at}\;\text{site}\;(\text{i},\text{j})\right)\right].$$ (29)

### Impact of antivirals on HSV-2 infection and lesion development

To account for the effects of the HSV-2 antiviral drug pritelivir in the mathematical model designed to describe chronic HSV-2 infection in the genital mucosa, we include a new parameter *ζ* to describe the effectiveness of the antiviral in suppressing HSV-2 replication. Here, *ζ* can take values in [0, 1] with 0 representing an antiviral dosage that has no effect on HSV-2 replication and 1 representing complete suppression of replication. As pritelivir is a helicase-primase inhibitor that suppresses viral replication, we assume this suppression decreases both the amount of virus that enters the simulation region from the neurons, and the amount of virus an infected epithelial cell produces; this replaces rates *ϕ* and *p* in the model with *ϕ*(1 − *ζ*) and *p*(1 − *ζ*) respectively.

We analyzed four potential values for *ζ* (*ζ* = 0.15, *ζ* = 0.5, *ζ* = 0.7, *ζ* = 0.85) and studied the resulting effects on lesion dynamics. Previous analysis predicts these *ζ* values correspond to doses of 10, 30, 55, and 80 mg of pritelivir a day [13]. These amounts all fall within the range of pritelivir doses given to patients in recent drug trials [31]. While other models have included the pharmacokinetics and pharmacodynamics of the drug [13], we more simply assume patients have a constant dosage within their genital mucosal tissue. This assumption can be considered acceptable due to pritelivir’s long 80-hour half-life which keeps drug conditions within the body relatively constant [13].

### HIV-1 infection probability for the entire genital region

To determine HIV-1 infection probability for the entire genital region, we assume that 22 of our 4cm^2^ simulation regions can be used to represent the entire area of the genital region, estimated to be 88 cm^2^ [30]. Within the genital tract of an HSV-2 infected individual, there may be multiple sites where different neurons are releasing HSV-2 into the genital tissue. Depending on the severity of HSV-2 infection, some of the 22 sites composing the entire genital tract will have dynamics given by our simulations while others can be considered “healthy”. We examined a range of different HSV-2 infection severities, varying the average number of sites centered around a single HSV-2 infected neuron. We chose to look at cases where patients have an average of 1, 2, or 3 sites of infection. This corresponds to HSV-2 neuronal release rates of 50, 100 and 150 virions/day/genital tract which have previously been found plausible [42].

We also incorporated the idea that sites of neuronal drip can move to different spots in the genital tract; at any given time, each of the 22 patches has a 1/22, 2/22, or 3/22 probability of being the site of neuronal drip, depending on the HSV-2 infection severity being examined. If acting as a site of neuronal drip, the state of that site was assumed to be described by a randomly chosen time point from one of the 50 full simulations of our model. Using the CD4+ T cell and tissue damage characteristics of each site, we determined the probability of HIV-1 infection at each of the 22 sites and then combined these using the equation

$$\begin{array}{c} \text{Prob(infection}\;\text{in}\;\text{vagina)}=1-\prod_{i=1}^{22}\left[1-\text{Prob(infection}\;\text{in}\;\text{sim.}\;\text{region}\;\text{i}\right)] \end{array}$$ (30)

to get the overall probability of HIV-1 infection per sexual act. This process was repeated 1000 times for each drug dose to produce distributions in infection probabilities.

### Parameters of the model

Due to recent interest in mathematically analyzing HSV-2 infections in the genital mucosa, many of the dynamics describing HSV-2 infection are well parameterized [7, 9, 11–13]. However, CD4+ T cell dynamics at the lesion site have not previously been examined from a mathematical standpoint. We therefore determined new values for the parameters governing CD4+ T cell behaviour in the genital epithelium. One challenge surrounding this task was the limited data regarding CD4+ T cell numbers in the genital mucosa. However, as CD4+ T cells are the main target of the HIV-1 virus, a representation of their dynamics is essential if we want to address questions related to HIV-1 infection. Fortunately, recent studies on the immune presence in the genital mucosa during different stages of herpetic lesion development reported both CD4+ and CD8+ T cell numbers at the lesion sites [4, 43].

A healthy individual without HSV-2 infection has approximately 68 CD4+ T cells per mm^2^ circulating around the epidermal layer of the genital epithelium [4]. Scaling this number to the 4 cm^2^ of our computational region indicates there should be 27200 CD4+ cells in the model when the patient is infection-free, directly corresponding to the fraction λ/*d*. Assuming the death rate, *d*, of CD4+ T cells is similar to that of CD8+ T cells, we set *d* = 0.07/day and λ = 1900/region-day to achieve the correct infection-free equilibrium value. With these two parameter values chosen, we can choose the parameters found in the expression for *X*_2_ (above), the rate of CD4+ T cell response to infection. A striking feature appearing in the experimental reports is the relatively unchanging ratio between CD4+ and CD8+ T cells [4, 43]. During the progression of an HSV-2 genital lesion, the CD4+ to CD8+ T cell ratio remains fairly constant, ranging from approximately 0.6 to 2.0 with a mean value of 1.06 in healthy tissue and 1.24 in HSV-infected tissue [4, 43]. As parameters describing CD8+ T cell dynamics are already known, we simply varied the parameters describing *X*_2_ until we reached a state where the CD4+ to CD8+ T cell ratio consistently fell within an appropriate range. In running fifty, one-year simulations of the full model including cytokines, with parameters of *r*_3_ = 42/day, *r*_4_ = 38/day, *θ*_1_ = 1.70/day, and *θ*_2_ = 1.40/day, the CD4+ to CD8+ ratio had an average of 1.4, ranging from 0.7-3.1. Since these ratios are similar to those observed experimentally, we used them for all simulations.

All parameter values of the model are recorded in Table 2.

**Table 2:** List of parameter values used in the model. Most parameter values were chosen to fall within the range of those found in the literature. Parameters without previously recorded values were estimated so that the model showed the expected dynamics. Parameter time units are in terms of days, while those dependent on space are scaled to be correct for a single 2 cm × 2 cm × 74 *μ*m simulation region.

Symbol	Description	Units	Value Chosen	Range in Literature	Citation
*g*	tissue repair rate	/day	0.22	0.22	[11]
*β*	infection rate	region/day	1.0 × 10^−7^	2.7 × 10^−9^ – 6.6 × 10^−7^	[7, 9, 11–13]
*a*	decay rate of *I*	/day	1.20	1.20 – 1.33	[7, 11]
*f*	death rate of *I* by *E*	region/day	0.010	0.001 – 0.200	[7, 11]
*r*_1_	*I* needed for half-max proliferation of *E*	/region	42	5 – 200	[7, 9, 11–13]
*r*_2_	*I* needed for half-max proliferation of *T*	/region	38	–	–
*r*_3_	*C* needed for half-max proliferation of *E*	/region	42	–	–
*r*_4_	*C* needed for half-max proliferation of *T*	/region	38	–	–
*θ*_1_	max proliferation rate of *E*	/day	1.70	0.98 – 7.20	[7, 9, 11–13]
*θ*_2_	max proliferation rate of *T*	/day	1.40	–	–
*δ*	decay rate of *E*	/day	0.05	6.64 × 10^−4^ – 8.30 × 10^−2^	[7, 9, 11–13]
*p*	release rate of *V* by *I*	/day	7.05 × 10^3^	10^3^ – 10^5^	[7, 9, 11–13]
*c*	decay rate of *V*	/day	8.8	6.2 – 96.0	[7, 9, 11–13, 44]
*ϕ*	rate of neuronal *V* drip	/region-day	50	1 – 2000	[7, 9, 11–13]
λ	rate of *T* inflow	/region-day	1900	–	–
*d*	decay rate of *T*	/day	0.07	–	–
*ω*_*V*_	motion coefficient of *V*	cm^2^/day	7.2 × 10^−4^	2.8 × 10^−6^ – 3.1 × 10^−3^	[45–47]
*ω*_*E*_, *ω*_*T*_	motion coefficient of *E* and *T*	cm^2^/day	7.2 × 10^−4^	1.3 × 10^−4^ – 1.4 × 10^−3^	[48, 49]
*ω*_*C*_	motion coefficient of *C*	cm^2^/day	2.45 × 10^−2^	1.30 × 10^−2^ – 8.64 × 10^−1^	[50, 51]
*m*	decay rate of *C*	/day	6.2	2.8 – 6.6	[50, 52]
*b*	production rate of *C* by *I*	/day	24.8	–	–
*n*	number of columns and rows in tissue grid	–	15	–	–
*E*_*ave*_	average concentration of *E*	/region	60,000	60, 000	[7]
*T*_*ave*_	average concentration of *T*	/region	50,000	50, 000	[4]
*H*_0_	concentration of *H* when tissue is healthy	/region	6 × 10^6^	6 × 10^6^	[7]

### Parameters for HIV-1

Many mathematical models have examined initial HIV-1 infection. However, few explicitly examined the dynamics occurring within the genital tissue, where HIV-1 infection is most often acquired. As HIV-1 and immune cell counts usually come from blood or plasma samples, the parameters of most models are fit to these numbers [25, 27, 28]. While dynamics occurring within the blood and epithelium may be similar, they may not be occurring at the same rates. While we were able to choose some parameter values based on those used in previous models, others were estimated based on our limited knowledge of infection behaviour in the female genital tract. This is with specific reference to the estimates for parameters *ψ* and *ℓ*. As only within-blood estimates have been recorded in the literature for these parameters, we chose values that corresponded with those fit for HSV-2 dynamics. Here, *ψ* matches with the rate of HSV-2 production and *ℓ* matches with the value for HSV-2 clearance. All HIV-1 parameters used in the model are listed in Table 3.

**Table 3:** List of parameter values used in the model to describe HIV-1 infection dynamics.

Symbol	Description	Units	Value Chosen	Range in Literature	Citation
*k*	infection rate of *T* by *P*	region/day	1 × 10^−7^	3.7 × 10^−8^ – 7.4 × 10^−4^	[25, 26]
*ψ*	release rate of *P* by *T*_2_	/day	7.05 × 10^3^	2 × 10^4^	[25]
*ℓ*	decay rate of *P*	/day	8.8	20 – 23	[25, 26]
*ω*_*P*_	motion coefficient of *P*	cm^2^/day	7.2 × 10^−4^	2.8 × 10^−6^ – 3.1 × 10^−3^	[45–47]
*η*	conversion rate of *T*_1_ to *T*_2_	/day	1	0.7 – 5	[25, 26]
*g*_1_	decay rate of *T*_1_	/day	0	0 – 0.5	[25, 26]
*g*_2_	decay rate of *T*_2_	/day	1.2	0.583 – 1	[25, 26]

## Supporting information

S1 Fig Spatial HSV-2 infection dynamics depicting lesion development when cytokines are not included. Stills of HSV-2 lesion development taken from a model simulation where the effects of cytokines were not included. Log_10_ HSV-2 counts (left), percents of tissue damage due to lesion development (centre), and log_10_ CD8+ immune cell counts (right) are shown across various days as the lesion develops.
(TIFF)

S2 Fig Length of time required for an infection outcome to be decided following HIV-1 introduction. HIV-1 was introduced into 4 cm^2^ simulation regions modelling healthy tissue and various HSV-2 lesion stages. HSV-2 dynamics were paused until HIV-1 either went extinct or HIV-1 established a successful infection, defined as 8 or more infected cells. We show the times it took for infection decisions to be made for 20 000 simulations, at each HSV-2 infection stage. For simplicity, these simulations were run in a non-spatial version of the model. The number of simulations composing each box and whisker set are written within the box. As HSV-2 infection severity and initial HIV-1 inoculum increases, the time it takes for a successful infection to be established decreases. Reciprocally, the time it takes for an unsuccessful infection to go extinct increases. Median durations are marked by the lines passing through the boxes in the plot where the upper and lower quartiles are the top and bottom of the boxes. Whiskers show the maximum and minimum values, with outlier points also indicated as solid circles. We observe that in all cases, extinction almost always occurs within 1 day of infection, if it occurs. This time scale is a lot faster than the lesion time scale, justifying our decision to neglect lesion dynamics here. If, on the other hand, the infection is “successful” (reaches 8 infected cells), the decision can take longer, and the timing of the decision is influenced by the HSV-2 lesion stage. However, the vast majority of HIV-1 infection cases occur between peak lesion and 2 weeks after (categories 2-5) and these decisions are almost always made within 1-2 days of HIV-1 exposure. Again, this justifies our decision to neglect lesion dynamics in these cases. In the relatively rare cases of infection without a lesion, before peak lesion, or four weeks after (categories 0, 1 and 6) the time to definitive infection can be somewhat longer. When there is no lesion (case 0), there is obviously no need to include lesion dynamics. Similarly, four weeks after peak lesion (case 6), the lesion dynamics are rather slow-moving and so it is reasonable to ignore them. The slightly problematic case is when the HIV-1 infection event occurs 1 week prior to peak lesion (category 1 in the figure). Here, it does result that infection, if successful, can take 4-5 days to establish. Although we acknowledge this is not strictly accurate, we feel that such cases are so rare that we may neglect the potential impact of lesion dynamics during HIV-1 infection without substantially impacting our broader conclusions.
(TIFF)

S3 Fig Spatial correlation between CD4+ cell density and damaged tissue affect HIV-1 infection probability. The probability of HIV-1 infection following sexual exposure to semen containing different HIV-1 concentrations was examined in four scenarios. In all scenarios, the total number of CD4+ T cells and tissue damage remains the same (111907 cells and 27.8% of the region being lesioned); however, the scenarios vary in how the CD4+ cells and tissue damage are distributed within the simulation region. Scenario 1, where tissue damage and CD4+ cells are somewhat correlated, is an example distribution taken from our full simulations. Scenario 2 shows an artificial situation where tissue damage and CD4+ T cell density are perfectly correlated. Scenario 3 is an artificial situation where there is no correlation between tissue damage and CD4+ T cell density. Scenario 4 shows tissue damage and CD4+ cells uniformly distributed across the region. We find that HIV-1 infection risk is much higher when tissue damage and CD4+ cell density are well correlated. This result reinforces the importance of knowing the spatial composition of HSV-2 infected tissue and supports our use of an explicitly spatial model.
(TIF)
