# Supplementary figures and images for: Effects of spatiotemporal HSV-2 lesion dynamics and antiviral treatment on the risk of HIV-1 acquisition
Source: PLoS Comput Biol. 2018 Apr 26;14(4):e1006129. doi: 10.1371/journal.pcbi.1006129 (PMC5940244; doi:10.1371/journal.pcbi.1006129)

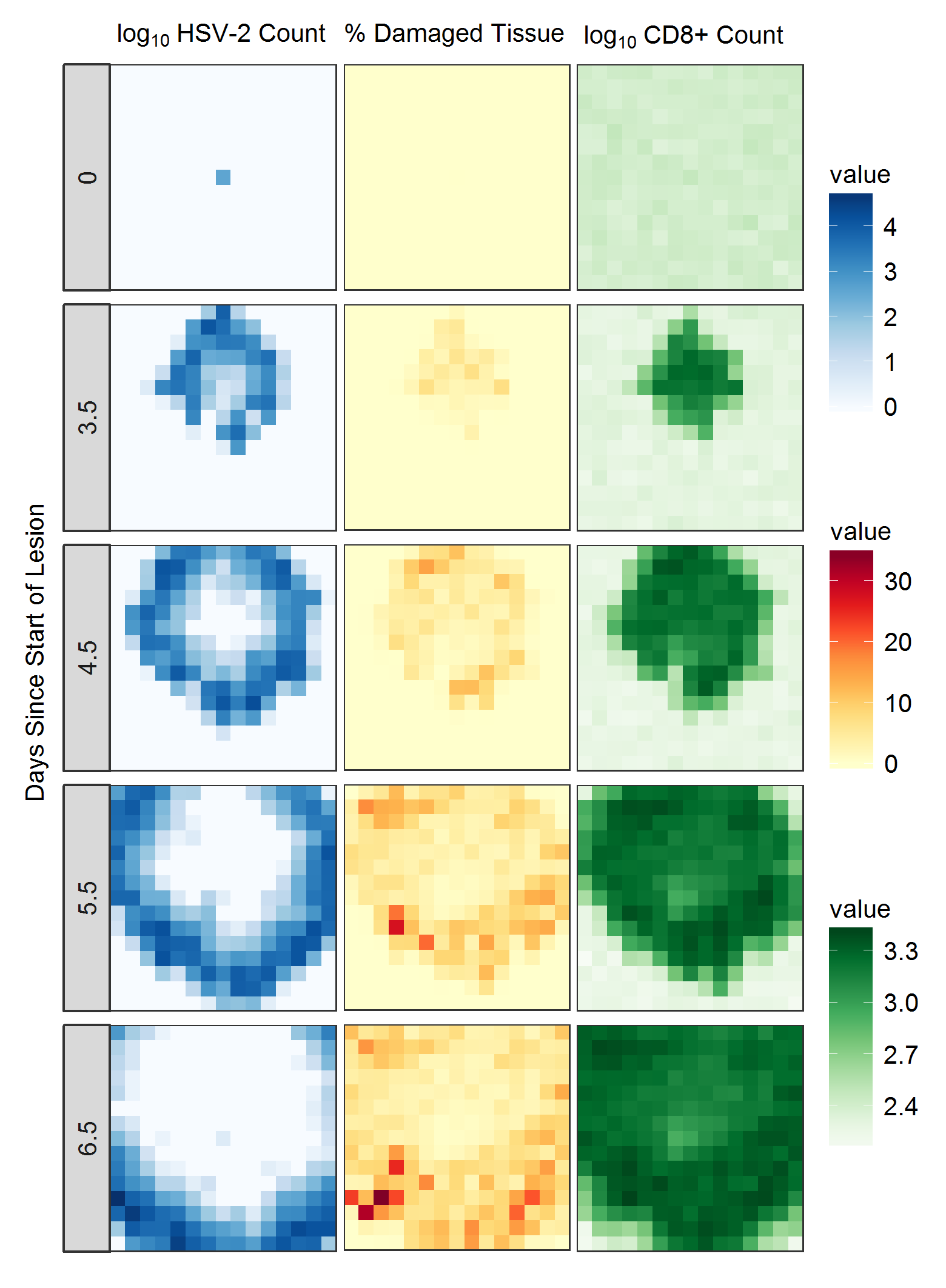

Supplement: S1 Fig — Stills of HSV-2 lesion development taken from a model simulation where the effects of cytokines were not included. Log10 HSV-2 counts (left), percents of tissue damage due to lesion development (centre), and log10 CD8+ immune cell counts (right) are shown across various days as the lesion develops. (TIFF) [file pcbi.1006129.s001.tiff]

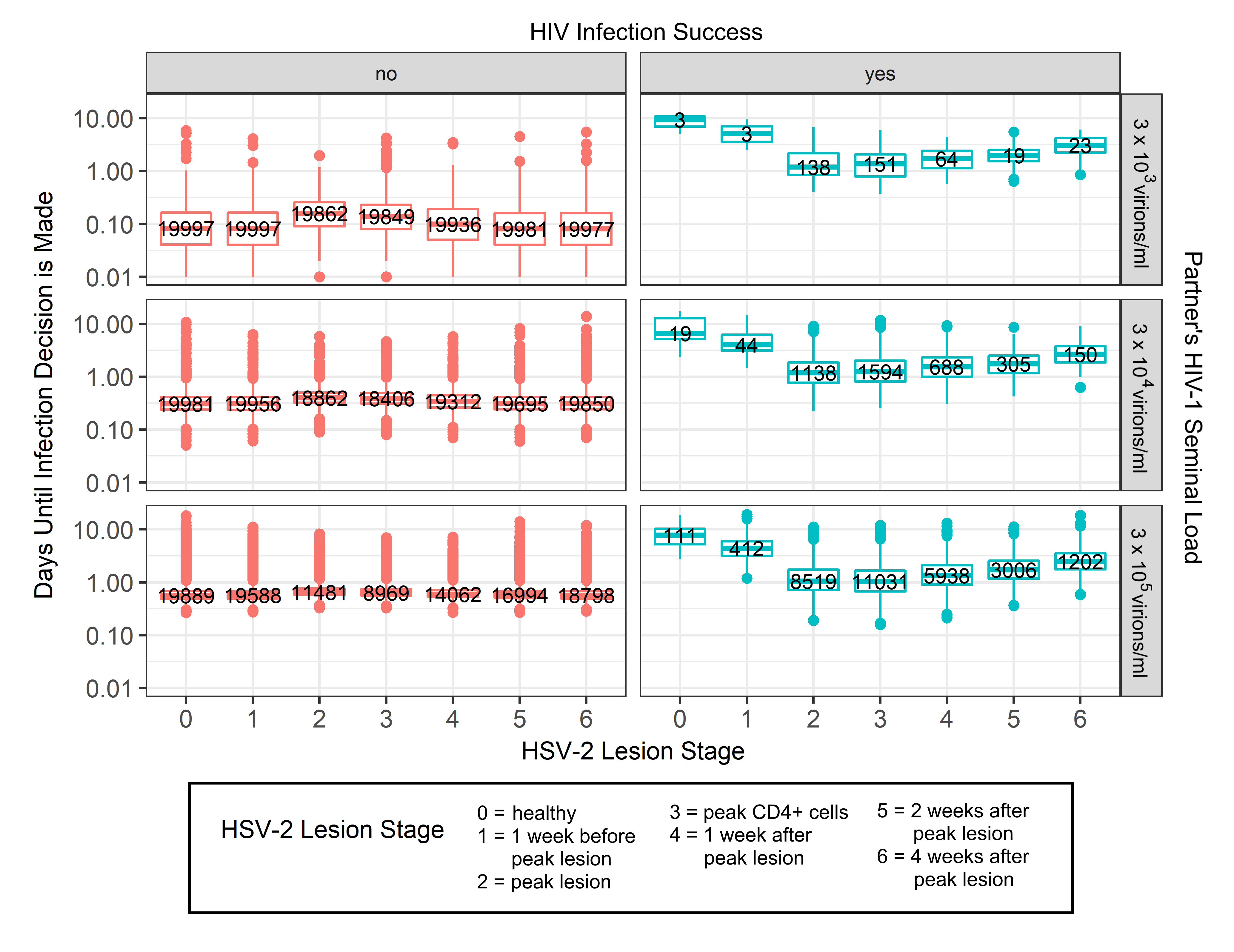

Supplement: S2 Fig — HIV-1 was introduced into 4 cm2 simulation regions modelling healthy tissue and various HSV-2 lesion stages. HSV-2 dynamics were paused until HIV-1 either went extinct or HIV-1 established a successful infection, defined as 8 or more infected cells. We show the times it took for infection decisions to be made for 20 000 simulations, at each HSV-2 infection stage. For simplicity, these simulations were run in a non-spatial version of the model. The number of simulations composing each box and whisker set are written within the box. As HSV-2 infection severity and initial HIV-1 inoculum increases, the time it takes for a successful infection to be established decreases. Reciprocally, the time it takes for an unsuccessful infection to go extinct increases. Median durations are marked by the lines passing through the boxes in the plot where the upper and lower quartiles are the top and bottom of the boxes. Whiskers show the maximum and minimum values, with outlier points also indicated as solid circles. We observe that in all cases, extinction almost always occurs within 1 day of infection, if it occurs. This time scale is a lot faster than the lesion time scale, justifying our decision to neglect lesion dynamics here. If, on the other hand, the infection is “successful” (reaches 8 infected cells), the decision can take longer, and the timing of the decision is influenced by the HSV-2 lesion stage. However, the vast majority of HIV-1 infection cases occur between peak lesion and 2 weeks after (categories 2-5) and these decisions are almost always made within 1-2 days of HIV-1 exposure. Again, this justifies our decision to neglect lesion dynamics in these cases. In the relatively rare cases of infection without a lesion, before peak lesion, or four weeks after (categories 0, 1 and 6) the time to definitive infection can be somewhat longer. When there is no lesion (case 0), there is obviously no need to include lesion dynamics. Similarly, four weeks after peak lesion (ca [file pcbi.1006129.s002.tiff]

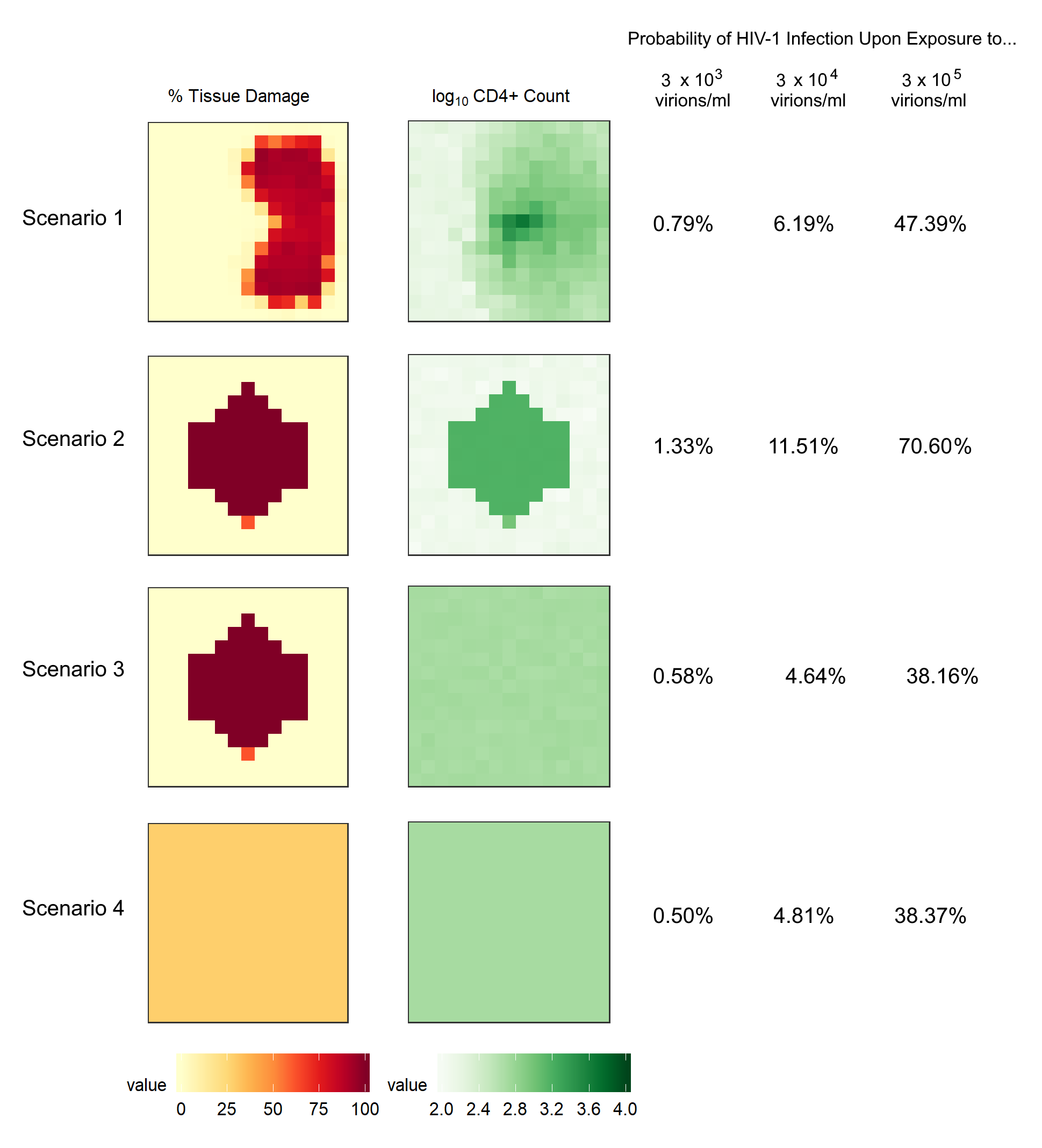

Supplement: S3 Fig — The probability of HIV-1 infection following sexual exposure to semen containing different HIV-1 concentrations was examined in four scenarios. In all scenarios, the total number of CD4+ T cells and tissue damage remains the same (111907 cells and 27.8% of the region being lesioned); however, the scenarios vary in how the CD4+ cells and tissue damage are distributed within the simulation region. Scenario 1, where tissue damage and CD4+ cells are somewhat correlated, is an example distribution taken from our full simulations. Scenario 2 shows an artificial situation where tissue damage and CD4+ T cell density are perfectly correlated. Scenario 3 is an artificial situation where there is no correlation between tissue damage and CD4+ T cell density. Scenario 4 shows tissue damage and CD4+ cells uniformly distributed across the region. We find that HIV-1 infection risk is much higher when tissue damage and CD4+ cell density are well correlated. This result reinforces the importance of knowing the spatial composition of HSV-2 infected tissue and supports our use of an explicitly spatial model. (TIF) [file pcbi.1006129.s003.tif]
